# Highly selective synthesis of d-amino acids via stereoinversion of corresponding counterpart by an in vivo cascade cell factory

**DOI:** 10.1186/s12934-020-01506-x

**Published:** 2021-01-09

**Authors:** Dan-Ping Zhang, Xiao-Ran Jing, Lun-Jie Wu, An-Wen Fan, Yao Nie, Yan Xu

**Affiliations:** 1 School of Biotechnology and Key laboratory of Industrial Biotechnology, Ministry of Education, Jiangnan University, 1800 Lihu Road, Wuxi, 214122 China; 2 State Key Laboratory of Food Science and Technology, Jiangnan University, Wuxi, 214122 China; 3 Suqian Industrial Technology Research Institute of Jiangnan University, Suqian, 223814 China

**Keywords:** d-amino acids, In vivo, Cascade, Whole-cell biocatalysis, Stereoinversion, Selectivity

## Abstract

**Background:**

d-Amino acids are increasingly used as building blocks to produce pharmaceuticals and fine chemicals. However, establishing a universal biocatalyst for the general synthesis of d-amino acids from cheap and readily available precursors with few by-products is challenging. In this study, we developed an efficient in vivo biocatalysis system for the synthesis of d-amino acids from l-amino acids by the co-expression of membrane-associated l-amino acid deaminase obtained from *Proteus mirabilis* (LAAD), *meso*-diaminopimelate dehydrogenases obtained from *Symbiobacterium thermophilum* (DAPDH), and formate dehydrogenase obtained from *Burkholderia stabilis* (FDH), in recombinant *Escherichia coli*.

**Results:**

To generate the in vivo cascade system, three strategies were evaluated to regulate enzyme expression levels, including single-plasmid co-expression, double-plasmid co-expression, and double-plasmid MBP-fused co-expression. The double-plasmid MBP-fused co-expression strain *Escherichia coli* pET-21b-MBP-*laad*/pET-28a-*dapdh*-*fdh*, exhibiting high catalytic efficiency, was selected. Under optimal conditions, 75 mg/mL of *E. coli* pET-21b-MBP-*laad*/pET-28a-*dapdh*-*fdh* whole-cell biocatalyst asymmetrically catalyzed the stereoinversion of 150 mM l-Phe to d-Phe, with quantitative yields of over 99% *ee* in 24 h, by the addition of 15 mM NADP^+^ and 300 mM ammonium formate. In addition, the whole-cell biocatalyst was used to successfully stereoinvert a variety of aromatic and aliphatic l-amino acids to their corresponding d-amino acids.

**Conclusions:**

The newly constructed in vivo cascade biocatalysis system was effective for the highly selective synthesis of d-amino acids via stereoinversion.

## Background

d-Amino acids, as chiral auxiliaries and chiral synthons in organic synthesis, play important roles in the production of pharmaceuticals and fine chemicals [1–3]. For example, they are key components in β-lactam antibiotics, fertility drugs, anticoagulants, and pesticides [1, 4, 5]. Various methods have been developed for the synthesis of d-amino acids. These methods can be categorized into two fundamentally different routes: chemical and biocatalytic approaches [3, 6, 7]. Chemical methods generally synthesize d-amino acids by the chiral resolution of racemic d,l-amino acids or by asymmetric protocols from chiral or prochiral starting materials. However, high costs, low yields, low selectivity, and toxicity are major disadvantages of chemical methods due to d-amino acid racemization [6, 8]. There has been substantial progress in the development of biocatalytic methods in the past decade. Using enzymes as biocatalysts, d-amino acids can be produced under mild reaction conditions with high enantioselectivities, conversions, and space-time yields [9]. A number of enzymatic approaches have been used to produce d-amino acids, including the synthesis of d-amino acids from d-hydantoin catalyzed by d-hydantoinase coupled with d-carbamoylase [10], asymmetric reductive amination of an α-keto acid by d-amino acid dehydrogenase or d-amino acid aminotransferase [1, 11], hydrolysis of an *N*-acyl-d-amino acid by *N*-acyl-d-amino acid amidohydrolase [12, 13], and the kinetic resolution of a racemic mixture by l-amino acid oxidase [7]. However, these methods usually require specific substrates that are generally expensive and not commercially available. Considering that l-amino acids are mostly generated by fermentation from inexpensive and renewable natural sources, d-amino acid synthesis by stereoisomeric inversion with l-amino acids as the starting substrate provides an economic and effective approach. However, there are only few reports about the applicability of this approach [3].

Multi-enzyme cascade reactions have recently become a very important synthetic strategy in the field of biocatalysis. They have various advantages, including the lack of a need for laborious intermediate recovery as well as the availability of cheap substrates [14–17]. Cascade catalysis could be performed in vivo or in vitro [14, 17–19]. In vivo cascade biocatalysis systems mainly involve the co-expression of multiple enzymes in a single host [20, 21]. The construction of an enzymatic cascade within a living host cell offers many advantages over in vitro methods, since whole cells can be used without further processing (e.g., protein purification) prior to the biotransformation, in vivo biocatalysis would be a more effective and easy-to-use strategy than the in vitro enzyme preparation [14, 22]. In particular, when enzymes involved in the cascade route are membrane-associated and difficult to use freely in vitro, the in vivo cascade system is a good choice, and the cell-wall could protect the enzymes [23, 24]. As a powerful tool for the heterologous expression of various proteins, *Escherichia coli* is an ideal host for the development of whole-cell catalysts [17, 24, 25]. Multiple enzymes have been co-expressed in *E. coli* from one vector (polycistronic vector), from multiple vectors, or from a mixture [26]. A polycistronic vector generally consists of a cluster of genes under the control of a single strong promoter (e.g., T7 promoter), whereas each gene has its own ribosome-binding site (RBS) and a stop codon [21]. Owing to the advantages of the in vivo cascade biocatalysis system, the co-expression of multiple enzymes to set up an artificial reaction cascade in *E. coli* has become a powerful method to generate highly efficient designer cell catalysts [27]. Turner et al. co-expressed d-amino acid dehydrogenase (DAADH) from *Corynebacterium glutamicum* and glucose dehydrogenase (GDH) from *Bacillus megaterium,* in *E. coli,* for the synthesis of d-arylalanines from α-keto acids (with α-keto acid concentrations of 40 mM); d-arylalanines were produced in quantitative yields with > 98% *ee* [28]. Although these whole-cell reaction systems have been used to synthesize a variety of d-amino acids, they require expensive and commercially unavailable substrates and show low catalytic efficiency. So far, there are few reports on the synthesis of d-amino acids from l-amino acids by in vivo cascade whole-cell catalysts.

We previously constructed a biocatalytic cascade system for the asymmetric synthesis of d-amino acids by the stereoinversion of l-amino acids, using a combination of LAAD whole-cells (l-amino acid deaminase from *Proteus mirabilis*) (oxidative deamination module), DAPDH (H227V variant of *meso*-diaminopimelate dehydrogenases from *Symbiobacterium thermophilum*) (reductive amination module), and FDH (formate dehydrogenase from *Burkholderia stabilis*) (cofactor regeneration) [8, 29]. However, in this biocatalytic cascade system, the reaction intermediate needs to be transferred through the cell membrane for the connection between the two necessary steps of the stereoinversion reaction, which would affect the conversion efficiency of the entire biocatalytic system. In order to improve the catalytic efficiency of the cascade catalytic system, it would be necessary to develop an in vivo cascade cell factory for efficient asymmetric synthesis of d-amino acids by the stereoinversion of l-amino acids.

**Scheme 1:**
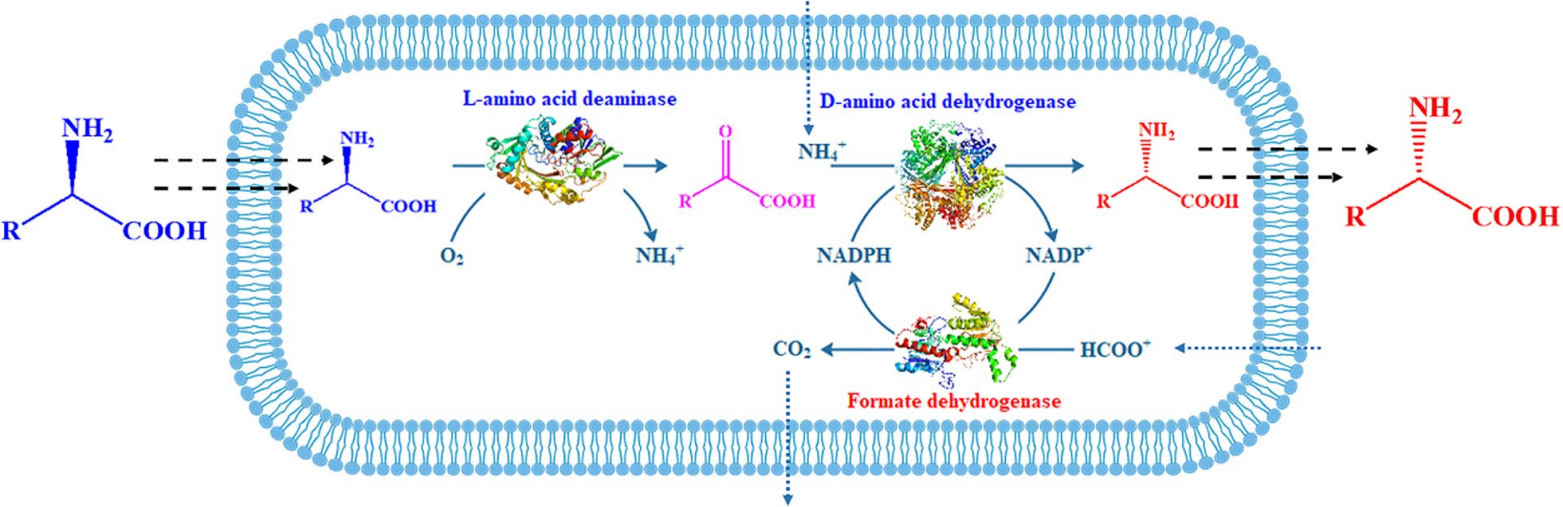
Scheme for d-amino acid production from l-amino acids using an in vivo cascade biocatalysis system by co-expressing l-amino acid deaminase, d-amino acid dehydrogenase, and formate dehydrogenase

In this study, *laad* encoding the l-amino acid deaminase, *dapdh* encoding the *meso*-diaminopimelate dehydrogenase, and *fdh* encoding formate dehydrogenase were co-expressed in *E. coli* for the asymmetric synthesis of d-amino acids via the stereoinversion of l-amino acids, as shown in Scheme 1. In the in vivo cascade catalytic system, LAAD catalyzing oxidative deamination and DAPDH catalyzing reductive amination were mainly used to catalyze the stereoinversion from l-amino acids to d-amino acids. By using only LAAD and DAPDH in the cells, it would be feasible to perform the stereoinversion transformation from l-amino acids to d-amino acids [8, 29]. Because DAPDH is NADPH dependent, however, FDH was used to construct the NADPH recycling system, improving the cofactor regeneration and the conversion efficiency of the entire system. To regulate the expression levels of the enzymes responsible for each reaction module, different plasmid-based co-expression systems involving RBSs, promoters, and fusion tags were constructed. Then, induction was optimized to further increase the expression of the three enzymes. Finally, the effects of the biocatalytic conditions of recombinant *E. coli* whole-cell biocatalysts were investigated. By using the obtained in vivo cascade cell factory, l-Phe was stereoinverted to d-Phe with high conversion efficiency and optical purity. Moreover, recombinant *E. coli* whole-cell biocatalysts also transformed a variety of aromatic and aliphatic l-amino acids into the corresponding d-amino acids.

## Results and discussion

### Construction of a multi-enzymatic cascade system

The co-expression of multiple enzymes in a single host by a shared protein synthesis machinery could decrease enzyme production costs compared to those for individual enzyme expression in multiple hosts followed by enzyme cocktailing [21]. However, the co-expression of multiple proteins may lead to a metabolic burden during cell growth, which can result in poor overexpression and thus impaired catalytic performance [21, 22, 30]. The implementation of an in vivo multi-enzyme cascade system in a designer cell catalyst requires the fine tuning of expression levels. The precise co-expression strategy affects the expression of target enzymes and the catalytic efficiency of the co-expression system [17, 21, 31]. As shown in Scheme 1, in our study, the designed in vivo cascade route was mainly composed of two modules: an oxidative deamination module catalyzed by l-amino acid deaminase and a reductive amination module catalyzed by d-amino acid dehydrogenase and formate dehydrogenase. To improve the overall catalytic efficiency of the co-expression system, three strategies were used to regulate the expression levels of the enzymes. (1) The three enzyme genes *laad*, *dapdh*, and *fdh*, which make up the oxidative deamination module (LAAD) and reductive amination modules (DAPDH and FDH), were cloned in a single pET-28a plasmid. Considering that the activity of LAAD was higher than that of DAPDH; accordingly, to enhance the expression of the DAPDH and FDH, the corresponding RBS sequences were added to *dapdh* and *fdh* [8]. To regulate and balance the expression intensity of the three enzyme genes, the positions of the two modules on the plasmid were adjusted. (2) The oxidative deamination module and reductive amination module were constructed on two different plasmids, pET-21b and pET-28a, respectively (pET-21b and pET-28a both contained the T7 promoter with ampicillin and kanamycin genes, respectively). In this way, the expression of the genes of the two modules would be regulated by the T7 promoter on the two plasmids for the independent expression of the two modules, thereby minimizing interactions among the three genes. (3) Since LAAD was a membrane-binding protein, an MBP-tag was added to the double-plasmid system for the improvement of soluble expression of LAAD. According to this expression strategy, four co-expression strains named *E. coli* pET-28a-*laad*-*dapdh*-*fdh*, *E. coli* pET-28a-*dapdh*-*fdh*-*laad*, *E. coli* pET-21b-*laad*/pET-28a-*dapdh*-*fdh*, and *E. coli* pET-21b-MBP-*laad*/pET-28a-*dapdh*-*fdh* were constructed.

**Fig. 1:**
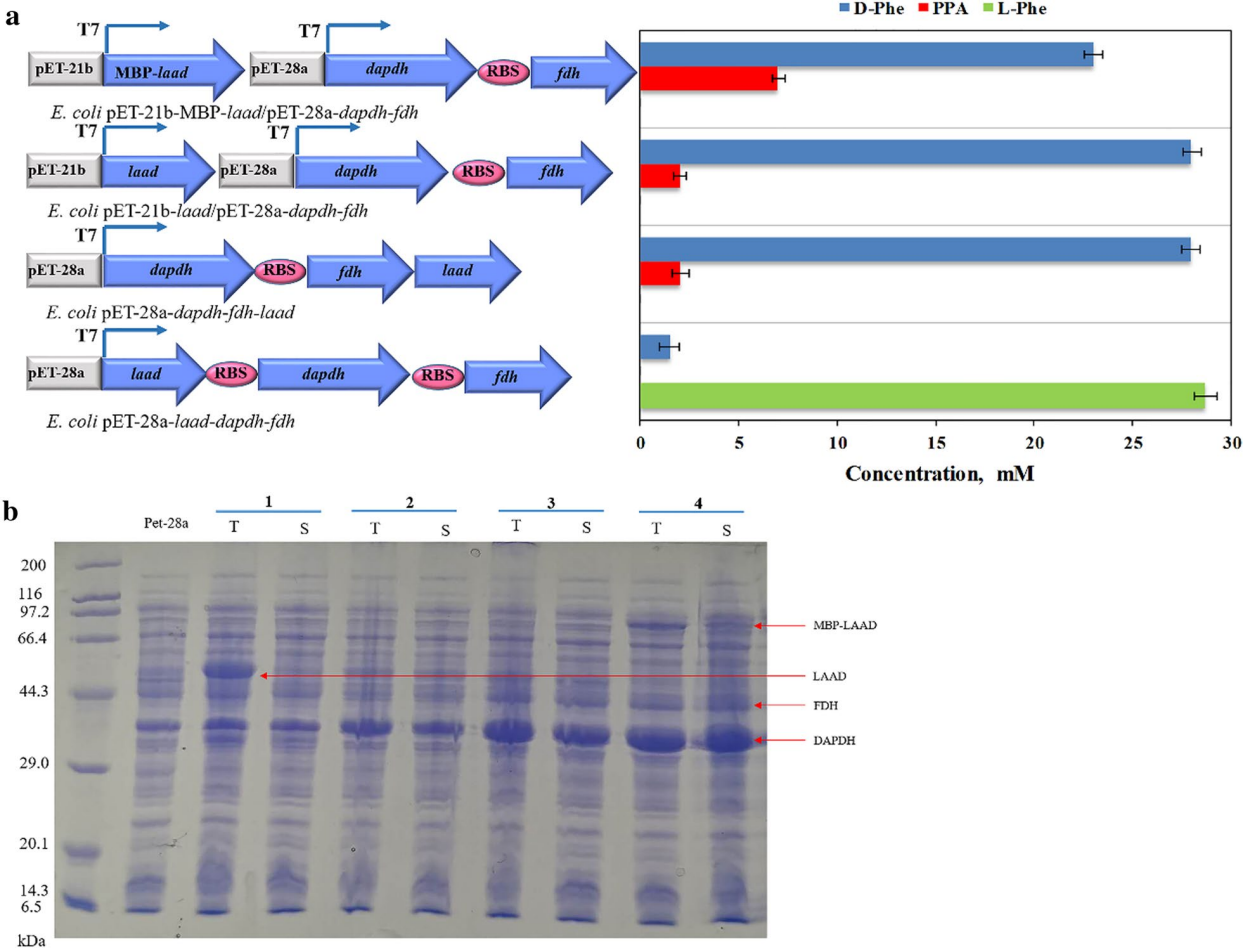
Construction of a multi-enzymatic cascade system by the regulation of enzyme expression. **a** Gene expression optimization with plasmids for the biotransformation of l-Phe into d-Phe. Reactions were carried out in Tris-HCl buffer (50 mM, pH 9.0) at 30 °C. The reaction mixture contained 50 mg/mL whole-cell biocatalyst, NADP^+^ (3 mM), NH_4_Cl (90 mM), sodium formate (60 mM), and l-Phe (30 mM). All reactions were carried out in Tris-HCl buffer (50 mM, pH 9.0) at 30 °C and 220 rpm. The values were averaged from triplicate measurements. **b** SDS-PAGE of recombinant *E. coli* pET-28a-*laad*-*dapdh*-*fdh* (1), *E. coli* pET28a-*dapdh*-*fdh*-*laad* (2), *E. coli* pET21b-*laad*/pET-28a-*dapdh*-*fdh* (3), and *E. coli* pET-21b-MBP-*laad*/pET-28a*-dapdh*-*fdh* (4). T: total cell lysate; S: soluble fraction. The samples were generated from 50 mg/mL *E. coli*

As shown in Fig. 1a, when the *laad*, *dapdh*, and *fdh* genes were cloned in the pET-28a plasmid, the position of *laad* had a great influence on the whole-cell catalyst. When *laad* was near the first position of the T7 promoter, although the expression of LAAD was very high, it mostly expressed as inclusion bodies, and DAPDH and FDH showed a certain degree of insoluble expression (Fig. 1b). Additionally, the catalytic activity of the *E. coli* pET-28a-*laad*-*dapdh*-*fdh* whole-cell biocatalyst was low (catalyzing 30 mM l-Phe), with only 1.5 mM concentration of the product, d-Phe. There is evidence that the closer a gene is to the end of a polycistronic operon, the lower is its expression [31]. Consequently, the first and the last positions in the polycistron would have the greatest impact on gene expression. Considering that LAAD is a membrane-binding protein, it may affect the expression of DAPDH and FDH when it is located near the first position of the T7 promoter. Therefore, in our study, the *laad* gene was placed at the end away from the T7 promoter. Surprisingly, all three enzymes exhibited normal expression, and the *E. coli* pET-28a-*dapdh*-*fdh*-*laad* whole-cell biocatalyst had high catalytic activity. The concentration of d-Phe in the reaction system was 28 mM, as shown in Fig. 1a and b. Subsequently, *laad*, *dapdh*, and *fdh* were constructed in two different plasmids, and the expression and catalytic efficiency of the three genes in the double-plasmid co-expression system were explored. When the three genes were co-expressed in two plasmids (*laad* or MBP-*laad* in the pET-21b plasmid, *dapdh* and *fdh* in the pET-28a plasmid, simultaneously), the two strains, *E. coli* pET-21b-MBP-*laad*/pET-28a-*stdapdh*-*fdh* and *E. coli* pET-21b-*laad*/pET-28a-*dapdh*-*fdh,* both had high catalytic activity; the concentrations of d-Phe in the reaction system were 23 and 28 mM. As shown in Fig. 1b, in addition, when the genes *laad*, *dapdh*, and *fdh* were cloned in pET-28a with *laad* at the first position close to the T7 promoter, the expression of *laad* increased. When *laad* was far from the T7 promoter, the expression of *dapdh* and *fdh* was enhanced while the expression of *laad* was weakened. In the double-plasmid co-expression system, the expression of *dapdh* and *fdh* was regulated by pET-28a separately. The expression of *dapdh* and *fdh* was enhanced while the expression of *laad* was weakened. Therefore, the expression of *laad* in pET-21b did not lead to the formation of an inclusion body as much as that in pET-28a when *laad* was placed at the first position. Consequently, we obtained three co-expression strains with high catalytic activity for subsequent analyses. The expression conditions and catalytic conditions of the strains were studied to further improve the catalytic efficiency, and thereby, obtain an optimized co-expression whole-cell biocatalyst.

### Effects of co-expression conditions on the catalytic efficiency

The effects of different expression conditions on the catalytic efficiency of recombinant strains were explored. As mentioned above, the substrate concentration was 30 mM, and the catalytic reactions were mainly conducted to measure the catalytic activities of the constructed whole-cell catalysts and to determine the feasibility of the in vivo cascade reaction. In order to further improve the catalytic efficiency, the optimization of the expression conditions of recombinant cells were conducted under a higher substrate concentration. Thus, the concentration of l-Phe was selected as 50 mM in the following studies.

**Fig. 2:**
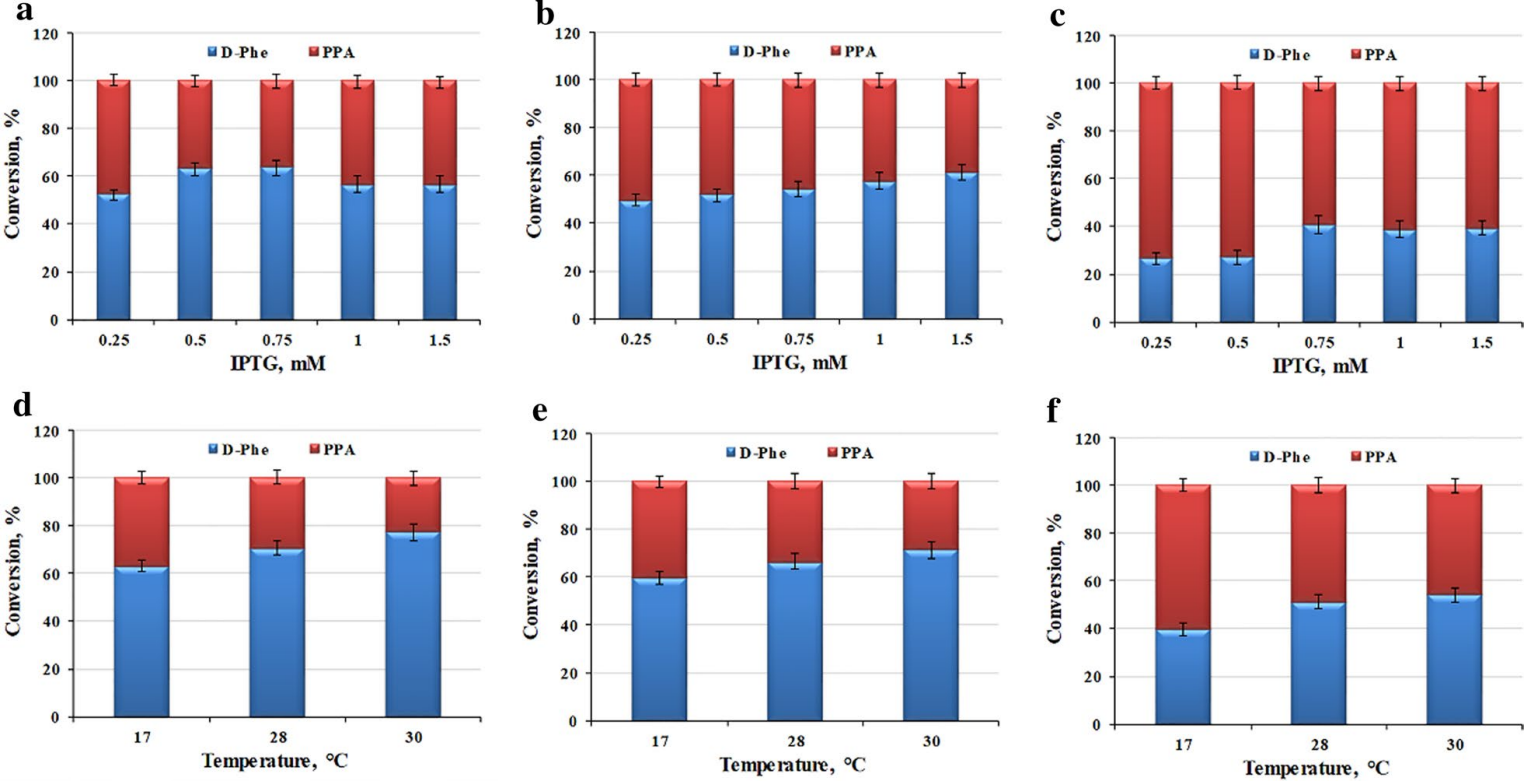
Effect of expression conditions on the catalytic efficiency of *E. coli* pET-21b-MBP*-laad*/pET-28a*-dapdh-fdh, E. coli* pET-21b*-laad/*pET-28a*-dapdh-fdh*, and *E. coli* pET-28a*-dapdh-fdh-laad*. **a** Effect of the IPTG concentration on the catalytic activity of *E. coli* pET-21b-MBP*-laad*/pET-28a*-dapdh-fdh*. The induction temperature was 17 °C. **b** Effect of the IPTG concentration on the catalytic activity of *E. coli* pET-21b*-laad/*pET-28a*-dapdh-fdh*. The induction temperature was 17 °C. **c** Effect of the IPTG concentration on the catalytic activity of *E. coli* pET-28a*-dapdh-fdh-laad*. The induction temperature was 17 °C. **d** Effect of the induction temperature on the catalytic activity of *E. coli* pET-21b-MBP*-laad*/pET-28a*-dapdh-fdh*. The IPTG concentration was 0.75 mM. **e** Effect of the induction temperature on the catalytic activity of *E. coli* pET-21b*-laad/*pET-28a*-dapdh-fdh*. The IPTG concentration was 1.5 mM. **f** Effect of the induction temperature on the catalytic activity of *E. coli* pET-28a*-dapdh-fdh-laad*. The IPTG concentration was 0.75 mM. The reaction mixture contained 50 mg/mL whole-cell biocatalyst, NADP^+^ (5 mM), NH_4_Cl (150 mM), sodium formate (100 mM) and l-Phe (50 mM). Reactions were all performed in Tris-HCl buffer (50 mM, pH 9.0) at 30 °C and 220 rpm for 6 h. The values were averaged from triplicate measurements

To optimize the protein expression conditions, the effects of the IPTG concentration and induction temperature on the biocatalyst activity of *E. coli* pET-21b-MBP-*laad*/pET-28a-*dapdh*-*fdh*, *E. coli* pET-21b-*laad*/pET-28a-*dapdh*-*fdh*, and *E. coli* pET-28a-*dapdh*-*fdh*-*laad* were studied. As shown in Fig. 2a, when the concentration of IPTG was 0.75 mM, the catalytic activity of the *E. coli* pET-21b-MBP-*laad*/pET-28a-*dapdh*-*fdh* whole-cell biocatalyst was the highest. Furthermore, higher concentrations of IPTG (1 and 1.5 mM) inhibited the catalytic activity of recombinant *E. coli* pET-21b-MBP-*laad*/pET-28a-*dapdh*-*fdh*. As determined by SDS-PAGE, all three proteins expressed inclusion bodies to some extent when the concentrations of IPTG were 1 and 1.5 mM (Additional file 1: Fig. S1). Therefore, the optimal IPTG concentration for *E. coli* pET-21b-MBP-*laad*/pET-28a-*dapdh*-*fdh* was 0.75 mM. Moreover, as shown in Fig. 2b and c, the optimal IPTG concentrations for *E. coli* pET-21b-*laad*/pET-28a-*dapdh*-*fdh* and *E. coli* pET28a-*dapdh*-*fdh*-*laad* were 1.5 and 0.75 mM, respectively. The effects of the IPTG concentration on the protein expression of *E. coli* pET-21b-*laad*/pET-28a-*dapdh*-*fdh* and *E. coli* pET-28a-*dapdh*-*fdh*-*laad* are summarized Additional file 1: Fig. S2–S3.

The effect of induction temperature on catalytic activity was further investigated. As shown in Fig. 2d–f, for all three co-expressed strains, the catalytic activity of the whole-cell biocatalyst increased as the induction temperature increased. When the induction temperature was 30 °C, the catalytic activity of the whole-cell biocatalyst was the highest. At the same time, under the optimal induction conditions, *E. coli* pET-21b-MBP-*laad*/pET-28a-*dapdh*-*fdh* had the highest catalytic activity (the optimal IPTG concentration was 0.75 mM and the optimal induction temperature was 30°C). According to our previous research, the fusion of the MBP-tag not only improves the soluble expression of the membrane-bound LAAD, but also increases its catalytic performance [29]. Therefore, in the *E. coli* pET-21b-MBP-*laad*/pET-28a-*dapdh*-*fdh* co-expression system, the improvement of the catalytic activity of LAAD increased the catalytic activity of the *E. coli* pET-21b-MBP-*laad*/pET-28a-*dapdh*-*fdh* whole-cell catalyst. Although the activity of LAAD was higher than that of the DAPDH, its active expression level was low due to the membrane-bound property [8]. The catalytic efficiency of the oxidative deamination module would be lower than that of the reductive amination module which drives the reaction balance towards the accumulation of target product, and the oxidative deamination module would be the rate-limiting step. Therefore, the catalytic performance of the whole-cell catalyst was improved after construct optimization involving RBSs, promoters, gene positions, and fusion tags. Consequently, the recombinant *E. coli* pET-21b-MBP-*laad*/pET-28a-*dapdh*-*fdh* was selected for further analyses.

### Effects of reaction components on catalytic efficiency

**Fig. 3:**
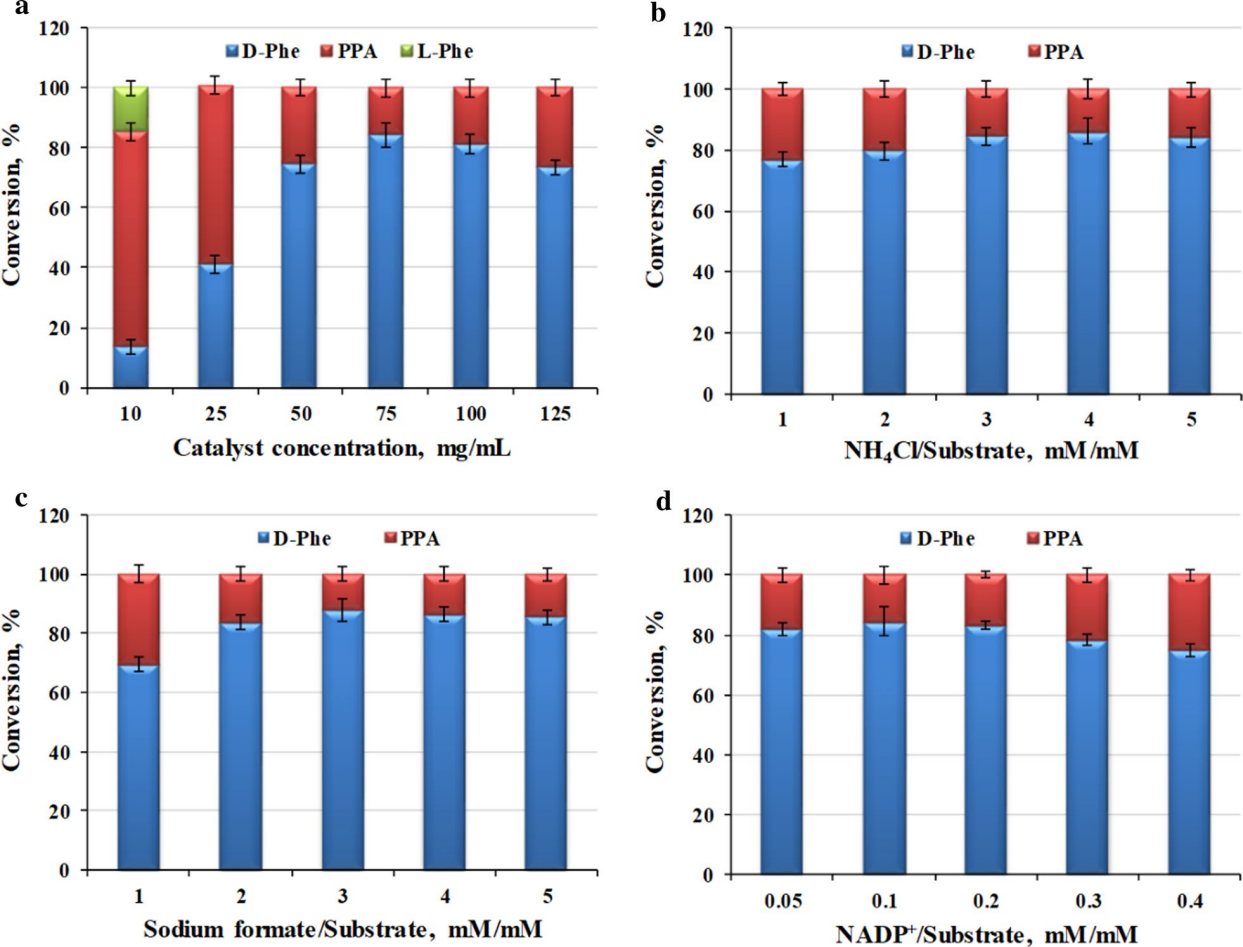
Effects of different concentrations of whole-cell biocatalyst, NH_4_Cl, sodium formate, and NADP^+^ on catalytic efficiency. **a** Effect of the whole-cell biocatalyst concentration on whole-cell catalytic activity. The concentration of NADP^+^ was 5 mM, the concentration of NH_4_Cl was 150 mM, and the concentration of sodium formate was 100 mM. **b** Effect of the NH_4_Cl concentration on whole-cell biocatalytic activity. The concentration of whole-cell biocatalyst was 75 mg/mL wet cells, the concentration of NADP^+^ was 5 mM, and the concentration of sodium formate was 100 mM. **c** Effect of the sodium formate concentration on whole-cell biocatalytic activity. The concentration of whole-cell biocatalyst was 75 mg/mL wet cells, the concentration of NADP^+^ was 5 mM, and the concentration of NH_4_Cl was 200 mM. **d** Effect of the NADP^+^ concentration on whole-cell biocatalytic activity. The concentration of whole-cell biocatalyst was 75 mg/mL wet cells, the concentration of NH_4_Cl was 200 mM, and the concentration of sodium formate was 150 mM. All reactions were carried out in Tris-HCl buffer (50 mM, pH 9.0) at 30°C and 220 rpm for 6 h. The values were averaged from triplicate measurements

To further improve the asymmetric production of d-Phe by whole-cell biocatalysis, the reaction conditions were optimized systematically. In our previous work of constructing in vitro cascade catalytic reaction system, the LAAD whole-cell biocatalyst and the enzyme DAPDH were more active at pH 9.0, which was favorable for the oxidative deamination module and the reductive amination module [8]. Therefore, in this work, the reaction pH value was conducted according to the previously optimized catalytic conditions for the cascade reaction. Concerning the reaction components, the effects of the concentration of the *E. coli* pET-21b-MBP-*laad*/pET-28a-*dapdh*-*fdh* whole-cell biocatalyst on conversion rates were firstly evaluated. The total conversion rate of d-Phe increased as the concentration of the whole-cell biocatalyst increased from 10 to 75 mg/mL wet cells (Fig. 3a). Moreover, the highest conversion rate of 84.5 % was achieved using 75 mg/mL wet cells after 6 h of transformation. As no obvious increase in the conversion rate was observed from 100 to 125 mg/mL wet cells, higher concentration of whole-cell catalyst might lead to the decrease of mass transfer rate in the reaction system, thus reducing the catalytic efficiency of whole-cell catalyst. Then, 75 mg/mL wet cells were chosen as the optimal amount for whole-cell transformation, and was used for subsequent experiments.

NH_4_Cl and sodium formate were used as ammonia donors and co-substrates in the previously constructed in vitro catalytic system. Although the NH_4_^+^ produced from LAAD could be recycled by DAPDH, the utilization rate of the generated NH_4_^+^ was very low. To guarantee the reaction efficiency, it would be necessary to add exogenous ammonia donor into the in vivo cascade catalytic system. Accordingly, the effects of NH_4_Cl and sodium formate concentrations on the catalytic activity of in vivo cascade system were investigated. As shown in Fig. 3b and c, the catalytic activity of the *E. coli* pET-21b-MBP-*laad*/pET-28a-*dapdh*-*fdh* whole-cell biocatalyst increased as the ratio of NH_4_Cl and soddium formate to substrate increased. When the concentration of NH_4_Cl was 4 times the concentration of the substrate and the concentration of sodium formate was 3 times the concentration of the substrate, the catalytic activity of whole-cell biocatalyst was the highest. The conversion rate did not increase obviously with the increase of the ratio of NH_4_Cl and soddium formate. Therefore, the optimal ratio of NH_4_Cl to substrate was 4.0, and the optimum amount of sodium formate was 3 times the substrate concentration.

Finally, the effect of the NADP^+^ concentration on the whole-cell biocatalyst efficiency was studied. As shown in Fig. 3d, 1-Phe was totally converted into phenylpyruvic acid (PPA) with the conversion reaching 100%; while the conversion from PPA into d-Phe was varied with the change of the NADP^+^ concentration. For the ratio of NADP^+^ to substrate below 0.2, the NADP^+^ concentration had a slight effect on the whole-cell biocatalyst efficiency with the conversion over 80%, and 0.1-fold NADP^+^ gave a high catalytic efficiency. However, for the ratio of NADP^+^ to substrate over 0.2, increasing the concentration of NADP^+^ reduced the catalytic efficiency of the whole-cell biocatalyst, and the conversion was only 74% when 0.4-fold NADP^+^ were added into the reaction system. Accordingly, the optimal concentration of NADP^+^ was 0.1 times the substrate concentration. During the reaction process, the cell membrane of the whole-cell catalyst might be destroyed to a certain extent, which would increase the permeability of the cell membrane and utilization of the exogenous NADP^+^ for cofactor regeneration in the cascade reaction [27, 32–34].

**Fig. 4:**
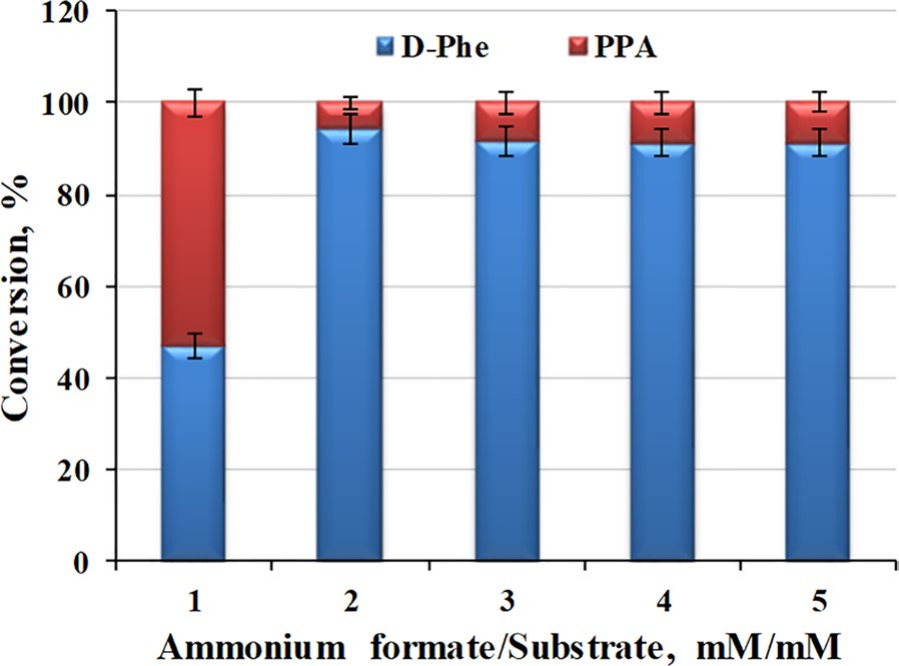
Effect of the ammonium formate concentration on whole-cell biocatalytic activity. The concentration of *E. coli* pET-21b-MBP*-laad*/pET-28a*-dapdh-fdh* wet cells was 75 mg/mL, the concentration of NADP^+^ was 5 mM, and the concentration of l-Phe was 50 mM. All reactions were carried out in Tris-HCl buffer (50 mM, pH 9.0) at 30 °C and 220 rpm for 6 h. The values were averaged from triplicate measurements

In the cascade catalytic route, NH_4_Cl and sodium formate are essential as an ammonia donor and co-substrate [8]. Ammonium formate could play dual roles as both a co-substrate and NH_4_^+^ donor for reductive amination. Therefore, ammonia formate was added to the reaction system to replace NH_4_Cl and sodium formate at a molar ratio of 1:1 to 5:1 (holding the concentration of substrate constant at 50 mM). As shown in Fig. 4, when the ratio of ammonium formate to substrate was 1:1, the conversion of d-Phe was 45 %. Whole-cell biocatalyst activity improved substantially in the presence of excess ammonium formate. The activity increased two-fold when the molar ratio was 2:1, probably because excess formate accelerated the reaction catalyzed by BsFDH and thus increased the efficiency of cofactor regeneration. Moreover, excess NH_4_^+^ could accelerate the reductive amination of PPA. However, no further increase in biocatalyst activity was detected when the molar ratio was further increased beyond 2:1. Consequently, the optimum molar ratio of ammonium formate to substrate for d-Phe production was 2:1, and this ratio was used in further experiments.

### Asymmetric synthesis of d-Phe from l-Phe

**Fig. 5:**
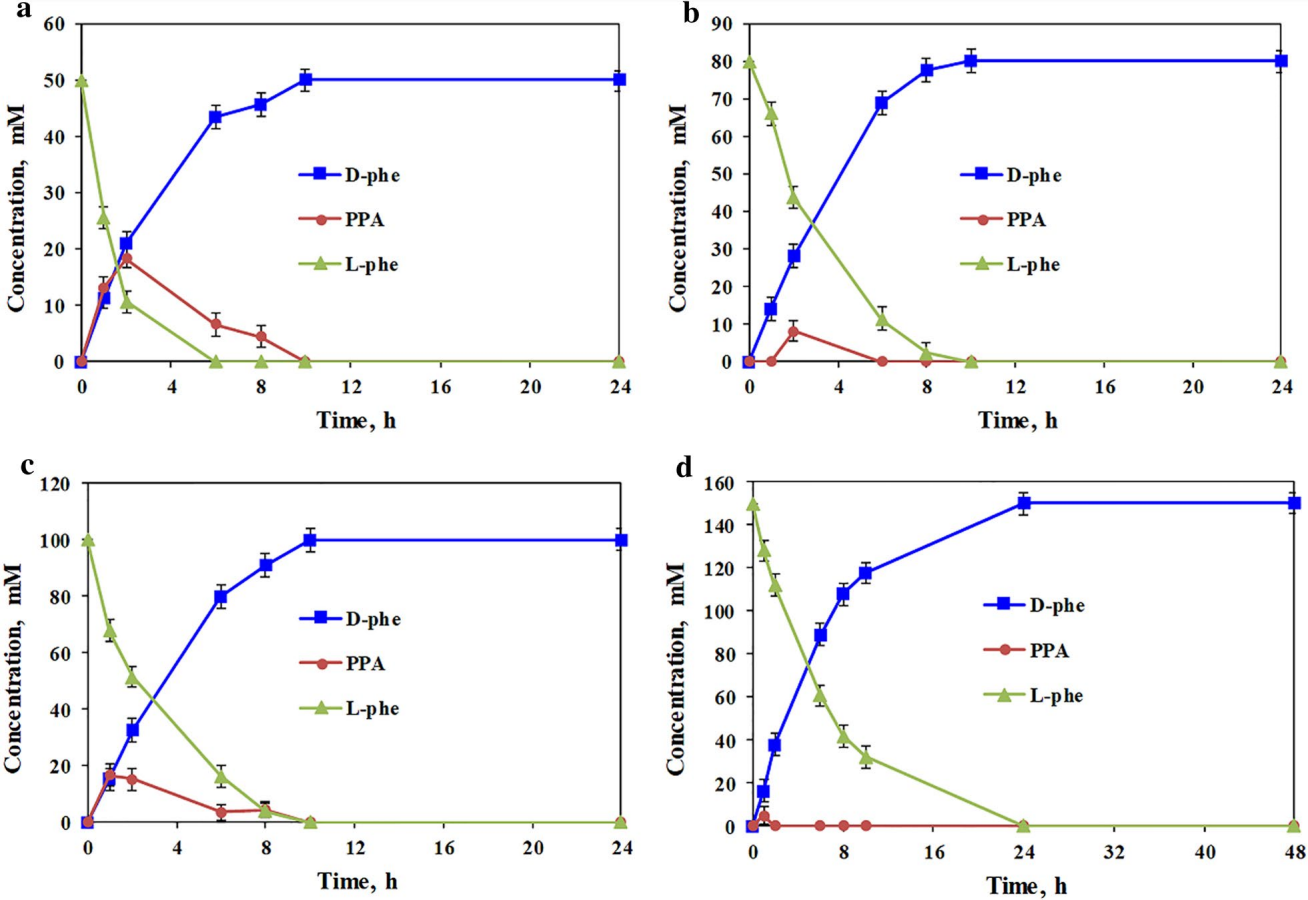
Time course of d-Phe production for various concentrations of l-Phe, including **a** 50 mM, **b** 80 mM, **c** 100 mM, and **d** 150 mM. The concentration of *E. coli* pET-21b-MBP*-laad*/pET-28a*-dapdh-fdh* wet cells was 75 mg/mL, the dosage of NADP^+^ was 0.1 times the substrate concentration, and the dosage of ammonium formate was twice the substrate concentration. All reactions were carried out in Tris-HCl buffer (50 mM, pH 9.0) at 30 °C and 220 rpm. The values were averaged from triplicate measurements

Based on the results of single-factor optimization experiments, the asymmetric reduction of l-Phe at substrate concentrations of 50–150 mM by *E. coli* pET-21b-MBP-*laad*/pET-28a-*dapdh*-*fdh* was performed, and the time courses of bioconversion are illustrated in Fig. 5. Ammonia formate at twice the concentration of the substrate, was added to the reaction system as an ammonia donor and co-substrate. When the concentration of l-Phe was 50–100 mM, the substrate was completely converted within 10 h. In the early stage of the reaction, there was a certain degree of accumulation of the intermediate product PPA, but it was quickly converted into the final product. However, further increasing the l-Phe concentration to 150 mM completely transformed the substrate into d-Phe within 24 h. There was almost no accumulation of the intermediate product, PPA, in the reaction process. This may be explained by the high concentration of the substrate, l-Phe, which can inhibit the catalytic efficiency of LAAD to a certain extent, thereby reducing the output of intermediate products, with rapid conversion by DAPDH. When NH_4_Cl and sodium formate were used as an ammonia donor and co-substrate, respectively, *E. coli* pET-21b-MBP-*laad*/pET-28a-*dapdh*-*fdh* catalyzed 100 and 150 mM l-Phe, with conversion rates of 82.3% and 59.5% after 48 h, respectively (Additional file 1: Fig. S4A and B). This is consistent with previous results for the in vitro cascade system with NH_4_Cl and sodium formate as the ammonia donor and co-substrate [8]. Therefore, the addition of ammonia formate could improve the catalytic efficiency of the *E. coli* pET-21b-MBP-*laad*/pET-28a-*dapdh*-*fdh* whole cell biocatalyst. By using the obtained in vivo cascade cell factory *E. coli* pET-21b-MBP-*laad*/pET-28a-*dapdh*-*fdh*, consequently, 150 mM l-Phe was stereoinverted to d-Phe with high conversion efficiency and optical purity (conversion rate, 100%; *ee* > 99%), giving nearly 1.9 times increase of the substrate concentration compared with the previous in vitro cascade system. Therefore, this in vivo cascade catalytic system constructed in this work performed a high catalytic efficiency for the stereoinversion of l-amino acids under an increased substrate concentration. In addition, the ammonia formate concentration only needed to be twice the substrate concentration, improving the catalytic efficiency while reducing costs, thereby conforming to the view of green chemistry.

### Asymmetric synthesis of d-amino acids from corresponding counterparts

The applicability of the *E. coli* pET-21b-MBP-*laad*/pET-28a-*dapdh*-*fdh* whole-cell biocatalyst for the asymmetric conversion of a variety of natural and noncanonical l-amino acids into the corresponding d-amino acids was evaluated. As shown in Table 1, the l-amino acids (30 mM) were mostly converted into the desired d-amino acids. For substrates with bulky groups, such as l-Phe, l-homophenylalanine, 2-chloro-l-phenylalanine, 3-chloro-l-phenylalanine and 4-chloro-l-phenylalanine, which are aromatic amino acids, the *E. coli* pET-21b-MBP-*laad*/pET-28a-*dapdh*-*fdh* whole-cell biocatalyst produced the corresponding d-amino acids with a high conversion rate (100 %) and optical purity (> 99% *ee*). In addition, the whole-cell biocatalyst could efficiently convert aliphatic amino acids, such as l-leucine, l-norvaline, l-glutamic acid, and l-methionine, to the corresponding d-amino acids, with d-leucine, d-glutamic acid, d-methionine and d-norvaline obtained in quantitative conversion and > 99% *ee*. In particular, when the substrates were l-glutamic acid and l-methionine, the conversion rates were 100% and the optical purities were > 99% *ee*. It also could be observed that the in vivo cascade catalytic system catalyzed limited aliphatic l-amino acid in a high conversion efficiency, which might be due to somewhat low activity of LAAD and DAPDH towards certain kinds of substrates. These results were consistent with those for the in vitro cascade catalytic system in our previous study [8]. Consequently, the *E. coli* pET-21b-MBP-*laad*/pET-28a-*dapdh*-*fdh* whole-cell biocatalyst could catalyze a variety of natural and noncanonical l-amino acids with high substrate concentration and high efficiency. Therefore, the in vivo multi-enzyme cascade system is efficient and applicable for the asymmetric synthesis of d-amino acids via stereoinversion.

**Table 1:** Stereoinversion rates of various l-amino acids using *E. coli* pET-21b-MBP-*laad*/pET-28a-*dapdh*-*fdh*

Substrate	*E. coli* pET-21b-MBP-*laad*/pET-28a-*dapdh*-*fdh* whole-cell biocatalyst	
	Conversion (%)	Optical purity (% ee)^a^
l-Leucine	73.3	> 99
l-Glutamic acid	100	> 99
l-Lysine	51.5	
l-Methionine	100	> 99
l-Norvaline	61.8	> 99
l-Tyrosine	45.3	> 99
l-Phenylalanine	100	> 99
l-Tryptophan	44.6	
l-Homophenylalanine	100	> 99
2-Chloro-l-phenylalanine	100	> 99
3-Chloro-l-phenylalanine	100	> 99
4-Chloro-l-phenylalanine	100	> 99
4-Methyl-l-phenylalanine	67.4	

Reaction conditions: 30 mM substrate, 3 mM NADP^+^, 60 mM ammonium formate were added to 50 mM Tris-HCl buffer (pH 9.0). The concentration of *E. coli* pET-21b-MBP*-laad*/pET-28a*-dapdh-fdh* was 75 mg/mL wet cells. The reactions were carried out at 30 °C and 220 rpm for 24 h.

^a^Optical purity (%ee) was determined by HPLC after FDAA derivatization.

## Conclusions

In this study, LAAD, DAPDH, and FDH were assembled in a multi-enzyme cascade system in vivo. The double-plasmid co-expression strain, named *E. coli* pET-21b-MBP-*laad*/pET-28a-*dapdh*-*fdh,* was selected for further analyses owing to its high catalytic efficiency. After optimizing the reaction conditions for *E. coli* pET-21b-MBP-*laad*/pET-28a-*dapdh*-*fdh*, 150 mM l-Phe was quantitatively converted into d-Phe with greater than 99% *ee* in 24 h by the addition of a 0.1-fold substrate concentration of NADP^+^ and two-fold substrate concentration of ammonium formate. In particular, *E. coli* pET-21b-MBP-*laad*/pET-28a-*dapdh*-*fdh* efficiently converted a variety of readily available and inexpensive aromatic and aliphatic l-amino acids into the corresponding d-amino acids. This newly constructed in vivo multi-enzyme cascade system is a promising, sustainable, and cost-efficient approach for the synthesis of a broad range of d-amino acids.

## Methods

### Materials

The *laad* gene from *P. mirabilis* (GenBank accession no. EU669819.1), the *dapdh* gene from *S. thermophilum* (GenBank accession no. BAD40410.1), and *fdh* from *B. stabilis* (GenBank accession no. ACF35003.1) were stored in our laboratory [8, 29, 35–38]. Enzymes, vectors, oligonucleotides, and other reagents for DNA cloning and amplification were obtained from Takara-Bio Co. (Kusatsu, Japan) and Novagen Co. (Madison, WI, USA). The l-amino acids and d-amino acids were purchased from Sinopharm Chemical Reagent Co., Ltd. (Shanghai, China). NADP^+^ was purchased from Sigma-Aldrich (St. Louis, MO, USA). Acetonitrile and methanol for high-performance liquid chromatography (HPLC) were of chromatographic grade (Honeywell Co., Charlotte, NC, USA). All other chemicals were of analytical grade and commercially available.

### Construction of the multi-enzymatic cascade system

Primers used in this study were listed in Additional file 1: Table S1. To construct *E. coli* pET-28a-*laad*-*dapdh*-*fdh*, the *laad* gene, *dapdh* gene, and *fdh* gene were cloned in pET-28a by homologous recombination. The RBSs for *dapdh* and *fdh* were calculated using the Salis Lab RBS Calculator and the translation initiation rates were set to 100% (https://salislab.net/software/doLogin). Then, the plasmid pET-28a-*laad*-*dapdh*-*fdh* was transformed into *E. coli* BL21 (DE3) cells. After plating, individual colonies were picked and the plasmids were sequenced. To construct *E. coli* pET-28a-*dapdh*-*fdh*-*laad*, the *dapdh* gene, *fdh* gene, and *laad* gene were cloned into pET-28a by homologous recombination. Then, the plasmid pET-28a-*dapdh*-*fdh*-*laad* was transformed into *E. coli* BL21 (DE3) cells. After plating, individual colonies were picked and the plasmids were sequenced. To construct *E. coli* pET-21b-*laad*/pET-28a-*dapdh*-*fdh*, the *laad* gene was cloned into the plasmid pET-21b, and the *dapdh* gene and *fdh* gene were cloned into the plasmid pET-28a. Then, both pET-21b-*laad* and pET-28a-*dapdh*-*fdh* were transformed into *E. coli* BL21 (DE3) cells. Although both pET-28a and pET-21b employ pBR322 as origin, they contain kanamycin and ampicillin resistance genes, respectively. Under the selection pressure, the two plasmids can coexist stably in the same host cell [39]. After plating, individual colonies were picked and the plasmids were sequenced. To construct *E. coli* pET-21b-MBP-*laad*/pET-28a-*dapdh*-*fdh*, the *laad* gene was cloned in the plasmid pET-21b-MBP, and the *dapdh* gene and *fdh* gene were cloned in the plasmid pET-28a. Then, both pET-21b-MBP-*laad* and plasmid pET-28a-*dapdh*-*fdh* were transformed into *E. coli* BL21 (DE3) cells. After plating, individual colonies were picked and the plasmids were sequenced.

To investigate expression levels and catalytic efficiency, strains were propagated in 1 L of Luria–Bertani medium containing 100 mg/mL kanamycin (or both 100 mg/mL kanamycin and 100 mg/mL ampicillin) at 37 °C. The culture was induced by the addition of isopropyl β-d-1-thiogalactopyranoside (IPTG) to a final concentration of 0.5 mM when the optical density (λ  =  600 nm) was 0.6–0.8, after which it was incubated for an additional 16 h at 17 °C at 200 rpm. After centrifugation at 6000×*g* and 4 °C for 20 min, the recombinant cells were washed with potassium phosphate buffer. To test the catalytic activity of co-expression recombinant cells, 50 mg/mL whole-cell biocatalyst, 3 mM NADP^+^, 90 mM NH_4_Cl, 60 mM sodium formate, and 30 mM l-Phe were added to 2 mL of Tris-HCl buffer (50 mM, pH 9.0). After reaction at 30 °C and 220 rpm for 6 h, an equal volume of 10 % (w/v) trichloroacetic acid solution was added to stop the reaction. The mixture was centrifuged at 12000×*g* for 5 min. The amount of PPA generated in the reaction process was determined using ferric chloride solution [8, 29, 40] and the amounts of l-Phe and d-Phe were determined by HPLC after derivation with 1-fluor-2,4-dinitrophenyl-5-l-alanine amide (FDAA) [2, 8, 29].

### Effects of co-expression conditions on catalytic efficiency

To investigate the effect of induction conditions on *E. coli* pET-21b-MBP-*laad*/pET-28a-*dapdh*-*fdh*, *E. coli* pET-21b-*laad*/pET-28a-*dapdh*-*fdh,* and *E. coli* pET-28a-*dapdh*-*bsfdh*-*laad* whole cell biocatalytic activity, the three co-expression strains were propagated in 50 mL of Luria–Bertani medium containing 100 mg/mL kanamycin (or 100 mg/mL kanamycin and 100 mg/mL ampicillin) at 37 °C. For investigating the effect of IPTG concentration on three co-expression strains, the culture was induced by the addition of IPTG to a final concentration of 0.25–1.5 mM when the optical density (λ  =  600 nm) was 0.6–0.8, after which it was incubated for an additional 16 h at 17 °C at 200 rpm. For investigating the effect of induction temperature on three co-expression strains, the concentration of IPTG used to induce the expression of *E. coli* pET-21b-MBP-*laad*/pET-28a-*dapdh*-*fdh*, *E. coli* pET-21b-*laad*/pET-28a-*dapdh*-*fdh,* and *E. coli* pET-28a-*dapdh*-*fdh*-*laad* were 0.75, 1.5, and 0.75 mM, respectively. When the optical density (λ  = 600 nm) was 0.6–0.8, after which it was incubated for an additional 16 h at 17°C, 28°C, and 30 °C at 200 rpm. After centrifugation at 6000×*g* and 4 °C for 20 min, the recombinant cells were washed with potassium phosphate buffer. To test the catalytic activity of co-expression recombinant cells, 50 mg/mL whole-cell biocatalyst, 5 mM NADP^+^, 150 mM NH_4_Cl, 100 mM sodium formate, and 50 mM l-Phe were added to 2 mL of Tris-HCl buffer (50 mM, pH 9.0). After reaction at 30 °C and 220 rpm for 6 h, an equal volume of 10 % (w/v) trichloroacetic acid solution was added to stop the reaction. The mixture was centrifuged at 12000×*g* for 5 min. The amount of PPA generated in the reaction process was determined using ferric chloride solution, and the amounts of l-Phe and d-Phe were determined by HPLC after derivation with FDAA.

### Effects of reaction components on catalytic efficiency

*E. coli* pET-21b-MBP-*laad*/pET-28a-*dapdh*-*fdh* whole-cell biocatalysts were propagated in 1 L of Luria–Bertani medium containing 100 mg/mL kanamycin and 100 mg/mL ampicillin at 37 °C. The culture was induced by the addition of IPTG to a final concentration of 0.75 mM when the optical density (λ  =  600 nm) was 0.6–0.8, following by incubation for an additional 16 h at 30 °C at 200 rpm. After centrifugation at 6000×*g* and 4 °C for 20 min, the recombinant cells were washed with potassium phosphate buffer. Then, the *E. coli* pET-21b-MBP-*laad*/pET-28a-*dapdh*-*fdh* whole-cell biocatalysts were used to catalyze the conversion of l-Phe to d-Phe. The reaction conditions were a whole-cell biocatalyst concentration of 10–125 mg/mL wet cells, NH_4_Cl concentration of 1–5 times the substrate concentration, sodium formate concentration of 1–5 times the substrate concentration, NADP^+^ concentration of 0.05–0.4 times the substrate concentration, and l-Phe concentration of 50 mM. The reaction buffer was Tris-HCl buffer (50 mM, pH 9.0). After reaction at 30°C and 220 rpm for 6 h, an equal volume of 10 % (w/v) trichloroacetic acid solution was added to stop the reaction. The mixture was centrifuged at 12000× *g* for 5 min. The amount of PPA generated in the reaction process was determined using ferric chloride solution, and the amounts of l-Phe and d-Phe were determined by HPLC after derivation with FDAA.

To investigate the effect of the ammonium formate concentration on *E. coli* pET-21b-MBP-*laad*/pET-28a-*dapdh*-*fdh* whole-cell catalytic activity, 75 mg/mL *E. coli* pET-21b-MBP-*laad*/pET-28a-*dapdh*-*fdh* whole-cell biocatalyst, 5 mM NADP^+^, ammonia formate concentration of 1-5 times the substrate concentration, and 50 mM l-Phe were added to 2 mL of Tris-HCl buffer (50 mM, pH 9.0) at 30 °C and 220 rpm for 6 h. Then, an equal volume of 10 % (w/v) trichloroacetic acid solution was added to stop the reaction. The mixture was centrifuged at 12000×*g* for 5 min. The amount of PPA generated in the reaction process was determined using ferric chloride solution, and the amounts of l-Phe and d-Phe were determined by HPLC after derivation with FDAA.

### Asymmetric synthesis of d-Phe from l-Phe

The reaction mixture was composed of 50, 80, 100, or 150 mM l-Phe, NADP^+^ at 0.1 times the substrate concentration, ammonia formate at 2 times the substrate concentration, and 75 mg/mL *E. coli* pET-21b-MBP-*laad*/pET-28a-*dapdh*-*fdh* whole-cell biocatalyst in 2 mL of Tris-HCl buffer (50 mM, pH 9.0). The reactions were carried out at 30°C and 220 rpm and were terminated by the addition of an equal volume of 10 % (w/v) trichloroacetic acid solution. The amount of PPA generated in the reaction process was determined using ferric chloride solution, and the amounts of l-Phe and d-Phe were determined by HPLC, after derivation with FDAA.

### Asymmetric synthesis of d-amino acids from corresponding counterparts

Various l-amino acids, including l-leucine, l-glutamic acid, l-lysine, l-methionine, l-norvaline, l-tyrosine, l-Phe, l-tryptophan, l-homophenylalanine, 2-chloro-l-phenylalanine, 3-chloro-l-phenylalanine, 4-chloro-l-phenylalanine, and 4-methyl-l-phenylalanine, were used as substrates for the asymmetric synthesis of the corresponding d-amino acids using the *E. coli* pET-21b-MBP-*laad*/pET-28a-*dapdh*-*fdh* whole-cell biocatalyst. The reaction mixture in 2 mL of Tris-HCl buffer (50 mM, pH 9.0) contained 30 mM substrate, 3 mM NADP^+^, 60 mM ammonia formate, and 75 mg/mL whole-cell biocatalyst. The reactions were carried out at 30 °C and 220 rpm for 24 h and were terminated by the addition of an equal volume of 10 % (w/v) trichloroacetic acid solution. The concentrations of l-amino acids and d-amino acids were determined by HPLC following FDAA derivation.

### Analytical methods

The amount of PPA in the reaction process was determined using ferric chloride solution [8, 41], which was prepared by dissolving 0.1 M ferric chloride in 6 mL of dimethyl sulfoxide, followed by the addition of 4 mL of deionized water and 200 μL of acetic acid, after which the solution was cooled in an ice bath. To determine the amount of PPA, a 15-μL aliquot of the reaction sample was added to 1 mL of the ferric chloride solution, and the mixture was incubated at 25°C for 2 min. The amount of PPA was immediately determined by measuring absorbance at 640 nm using a microplate reader.

The concentrations of l-amino acids and d-amino acids were determined by HPLC, following FDAA derivation. The FDAA reagent (Sigma-Aldrich) was used to produce diastereomeric derivatives of the amino acids. A 10 μL sample of the amino acid, 8 μL of 1 M NaHCO_3_, and 40 μL of 1 % (w/v) FDAA in acetone were mixed and heated for 1 h at 40°C. When the sample was cooled to room temperature, 8 μL of 1 N HCl and 934 μL of 40 % (v/v) aqueous acetonitrile were added to the mixture, followed by vortexing and filtering (0.22 μm) for HPLC [2, 8]. Samples were analyzed using a Develosil ODS-UG-5 column (150 mm  ×  4.6 mm). The HPLC conditions for analyzing phenylalanine were as follows: mobile phase A water (0.05% trifluoroacetic acid), mobile phase B acetonitrile (0.05% trifluoroacetic acid), with gradients from 20% B to 40% B over 40 min, and from 40% B to 50% B over 5 min at a flow rate of 1 mL/min, detection at 340 nm, 40 °C, and an injection volume of 20 μL (Additional file 1: Fig. S5). The HPLC conditions for analyzing other amino acids were as follows: mobile phase A 5 % acetonitrile (0.05 % trifluoroacetic acid, 1 % methanol), mobile phase B 60 % acetonitrile (0.05 % trifluoroacetic acid, 1 % methanol), linear gradient from 0 % B to 100 % B over 45 min at a flow rate 1 mL/min, injection volume of 20 μL (Additional file 1: Fig. S6–17) [8, 42].

## Supplementary Information

**Additional file 1: Table S1.** Primers used in this study. **Fig. S1.** SDS-PAGE analysis of *E. coli* pET-21b-MBP-laad/pET-28a-dapdh-fdh induced by different concentrations of IPTG. T: total cell lysate; S: soluble fraction. The samples were generated from 50 mg/mL of *E. coli* pET-21b-MBP-laad/pET-28a-dapdh-fdh cells. **Fig. S2.** SDS-PAGE analysis of *E. coli* pET-21b-laad/pET-28a-dapdh-fdh induced by different concentrations of IPTG. T: total cell lysate; S: soluble fraction. The samples were generated from 50 mg/mL of *E. coli* pET-21b-laad/pET-28a-dapdh-fdh cells. **Fig. S3.** SDS-PAGE analysis of *E. coli* pET-28a-dapdh-fdh-laad induced by different concentrations of IPTG. T: total cell lysate; S: soluble fraction. The samples were generated from 50 mg/mL of *E. coli*i pET-28a-dapdh-fdh-laad cells. **Fig. S4.** Time course of d-Phe production from diferent concentration of l-Phe. A: The concentration of l-Phe was 100 mM; B: The concentration of l-Phe was 150 mM. The concentration of *E. coli* pET-21b-MBP-laad/pET-28a-dapdh-fdh whole-cell biocatalyst was 75 mg/mL wet cell, the dosage of NADP+ was 0.1 time of substrate concentration, the dosage of NH4Cl was 4 times of the substrate concentration, and the dosage of sodium formate was 3 times of substrate concentration. All the reactions were carried out in Tris-HCl buffer (50 mM, pH 9.0) at 30 ℃ and 220 rpm. **Fig. S5.** HPLC analysis of l-Phe and d-Phe. Retention time of l-Phe is 34.620 min; retention time of d-Phe is 41.287 min. **Fig. S6.** HPLC analysis of l-leucine and d-leucine. Retention time of l-leucine is 32.313 min; retention time of d-leucine is 36.100 min. **Fig. S7.** HPLC analysis of l-glutamic acid and d-glutamic acid. Retention time of l-glutamic acid is 18.760 min; retention time of d-glutamic acid is 20.327 min. **Fig. S8.** HPLC analysis of l-lysine and d-lysine. Retention time of l-lysine is 28.267 min; retention time of d-lysine is 34.46 min. **Fig. S9.** HPLC analysis of l-methionine and d-methionine. Retention time of l-methionine is 28.54 min; retention time of d-methionine is 31.92 min. **Fig. S10.** HPLC analysis of l-norvaline and d-norvaline. Retention time of l-norvaline is 29.153 min; retention time of d-norvaline is 33.120 min. **Fig. S11.** HPLC analysis of l-tyrosine and D-tyrosine. Retention time of l-tyrosine is 37.067 min; retention time of d-tyrosine is 40.067 min. **Fig. S12.** HPLC analysis of l-tryptophan and D-tryptophan. Retention time of l-tryptophan is 30.773 min; retention time of l-tryptophan is 33.153 min. **Fig. S13.** HPLC analysis of l-homophenyalanine and d-homophenyalanine. Retention time of l-homophenyalanine is 35.04 min; retention time of d-homophenyalanine is 38.713 min. **Fig. S14.** HPLC analysis of 2-chloro-l-phenylalanine and 2-chloro-d-phenylalanine. Retention time of 2-chloro-l-phenylalanine is 37.66 min; retention time of 2-chloro-d-phenylalanine is 34.227 min. **Fig. S15.** HPLC analysis of 3-chloro-l-phenylalanine and 3-chloro-d-phenylalanine. Retention time of 3-chloro-l-phenylalanine is 35.473 min; retention time of 3-chloro-d-phenylalanine is 38.713 min. **Fig. S16.** HPLC analysis of 4-chloro-l-phenylalanine and 4-chloro-d-phenylalanine. Retention time of 4-chloro-l-phenylalanine is 36.12 min; retention time of 4-chloro-d-phenylalanine is 39.42 min. **Fig. S17.** HPLC analysis of 4-methyl-l-phenylalanine and 4-methyl-d-phenylalanine. Retention time of 4-methyl-l-phenylalanine is 35.347 min; retention time of 4-methyl-d-phenylalanine is 38.600 min.

## Data Availability

We declared that materials described in the manuscript, including all relevant raw data, will be freely available to any scientist wishing to use them for noncommercial purposes, without breaching participant confidentiality.
