# Supplementary material for: Highly selective synthesis of d-amino acids via stereoinversion of corresponding counterpart by an in vivo cascade cell factory
Source: Microb Cell Fact. 2021 Jan 9;20:11. doi: 10.1186/s12934-020-01506-x (PMC7797136; doi:10.1186/s12934-020-01506-x)
Supplement: Supplementary file 1 — Additional file 1: Table S1. Primers used in this study. Fig. S1. SDS-PAGE analysis of E. coli pET-21b-MBP-laad/pET-28a-dapdh-fdh induced by different concentrations of IPTG. T: total cell lysate; S: soluble fraction. The samples were generated from 50 mg/mL of E. coli pET-21b-MBP-laad/pET-28a-dapdh-fdh cells. Fig. S2. SDS-PAGE analysis of E. coli pET-21b-laad/pET-28a-dapdh-fdh induced by different concentrations of IPTG. T: total cell lysate; S: soluble fraction. The samples were generated from 50 mg/mL of E. coli pET-21b-laad/pET-28a-dapdh-fdh cells. Fig. S3. SDS-PAGE analysis of E. coli pET-28a-dapdh-fdh-laad induced by different concentrations of IPTG. T: total cell lysate; S: soluble fraction. The samples were generated from 50 mg/mL of E. colii pET-28a-dapdh-fdh-laad cells. Fig. S4. Time course of d-Phe production from diferent concentration of l-Phe. A: The concentration of l-Phe was 100 mM; B: The concentration of l-Phe was 150 mM. The concentration of E. coli pET-21b-MBP-laad/pET-28a-dapdh-fdh whole-cell biocatalyst was 75 mg/mL wet cell, the dosage of NADP+ was 0.1 time of substrate concentration, the dosage of NH4Cl was 4 times of the substrate concentration, and the dosage of sodium formate was 3 times of substrate concentration. All the reactions were carried out in Tris-HCl buffer (50 mM, pH 9.0) at 30 ℃ and 220 rpm. Fig. S5. HPLC analysis of l-Phe and d-Phe. Retention time of l-Phe is 34.620 min; retention time of d-Phe is 41.287 min. Fig. S6. HPLC analysis of l-leucine and d-leucine. Retention time of l-leucine is 32.313 min; retention time of d-leucine is 36.100 min. Fig. S7. HPLC analysis of l-glutamic acid and d-glutamic acid. Retention time of l-glutamic acid is 18.760 min; retention time of d-glutamic acid is 20.327 min. Fig. S8. HPLC analysis of l-lysine and d-lysine. Retention time of l-lysine is 28.267 min; retention time of d-lysine is 34.46 min. Fig. S9. HPLC analysis of l-methionine and d-methionine. Retention time of l-methionine is 28.54 [file 12934_2020_1506_MOESM1_ESM.docx]

**Supporting Information**

**Highly selective synthesis of d-amino acids via stereoinversion of corresponding counterpart by an *in vivo* cascade cell factory**

Dan-Ping Zhang^1^, Xiao-Ran Jing^1^, Lun-Jie Wu^1^, An-Wen Fan^1^, Yao Nie^1,3,*^, Yan Xu^1,2^

^1^ School of Biotechnology and Key laboratory of Industrial Biotechnology, Ministry of Education, Jiangnan University, Wuxi 214122, China.

^2^ State Key Laboratory of Food Science and Technology, Jiangnan University, Wuxi 214122, China.

^3^ Suqian Industrial Technology Research Institute of Jiangnan University, Suqian 223814, China

^*^ Correspondence: [ynie@jiangnan.edu.cn](mailto:ynie@jiangnan.edu.cn)

Key Laboratory of Industrial Biotechnology, Ministry of Education, School of Biotechnology, Jiangnan University, 1800 Lihu Road, Wuxi 214122, China

**Table S1** Primers used in this study

| Plasmid*^a^* | Primer (5’-3’)*^b^* |
| --- | --- |
| pET-28a-*laad*-*dapdh*-*fdh* | pm1-F: **TGGTGCCGCGCGGCAGC**CATATGATGGCAATAAGTAG |
|  | pm1-R: **CCTTATTTATTCTTAATCG**TTATCTGTTAGAAACGATACAGACTAAATGGT |
|  | st1-F: **CGATTAAGAATAAATAAGG**AGGTTTTTTATGGATAAATTACGTGTCGCG |
|  | st1-R: **CCTCCTTATTTGTCTTAC**CAAAATTGGCTTGACTAAACCAGCTTACGAATCCAC |
|  | bs1-F: **GTAAGACAAATAAGGAGG**TTTTTTAATGGCAACCGTGCTGTGCGTGC |
|  | bs1-R: **GTGGTGCTCGAGGGTTAA**ACGATAGCTCTGGGC |
|  | Vector1-F: **TTAACCCTCGAGCACCAC**CACCACCACCACTGAGATCCG |
|  | Vector1-R: **GCTGCCGCGCGGCACCA**GGCCGCTGCTGTGAT |
| pET-28a-*dapdh*-*fdh*-*laad* | st2-F: **CGCGGCAGCCATATGATG**GATAAATTACGTGTCGCGGTGGTGG |
|  | st2-R: **ACCTCCTTATTTGTCTTACC**AAAATTGGCTTGACTAAACCAGCTTACGAATCCACGCTTC |
|  | bs2-F: **GGTAAGACAAATAAGGAGGT**TTTTTAATGGCAACCGTGCTGTGCGTGCTG |
|  | bs2-R: **GGGTATATCTCCTTCTC**AGGTTAAACGATAGCTCTGGGCACCGGTA |
|  | pm2-F: **GAGAAGGAGATATACCC**ATATGATGGCAATAAGTAGAAGAAAATTTATT |
|  | pm2-R: **TGGTGGTGGTGCTCGAGG**AAACGATACAGACTAAATGGTTTGGCATCTA |
|  | Vector2-F: **CCTCGAGCACCACCACCA**CCACCACTGAGATCCGGC |
|  | Vector2-R: **CATCATATGGCTGCCGCG**CGGCACCAGGCCGCTG |
| pET-28a-*dapdh*-*fdh* | st3-F: **GCGGCAGCCATATGATGGAT**AAATTACGTGTCGCGGTGGTGGGTTACGG |
|  | st3-R: **CCTCCTTATTTGTCTTACC**AAAATTGGCTTGACTAAACCAGCTTACGAATCCACGCTTCACGGTC |
|  | bs3-F: **GGTAAGACAAATAAGGAGG**TTTTTTAATGGCAACCGTGCTGTGCGTGCTGTATCCG |
|  | bs3-R: **GGTGGTGCTCGAGTCAGGTT**AAACGATAGCTCTGGGCACCGGTACCAGCTA |
|  | Vector3-F: **AACCTGACTCGAGCACCACC**ACCACCACCACTGAGATCCGGCTGCTA |
|  | Vector3-R: **ATCCATCATATGGCTGCCGC**GCGGCACCAGGCCGCTGCTGTGAT |
| pET-21b-*laad* | pm3-F: **GAAGGAGATATACATATG**ATGGCAATAAGTAGAAGAAAATTTAT |
|  | pm3-R: **GGCCGCAAGCTTGTCGAC**TTAGAAACGATACAGACTAAATGG |
|  | Vector4-F: **GTCGACAAGCTTGCGGCC**GCACTCGAGCACCACC |
|  | Vector4-R: **CATATGTATATCTCCTTC**TTAAAGTTAAACAAAATT |
| pET-21b-MBP-*laad* | pm4-F: **CAGAGCCATATGATGGCAA**TAAGTAGAAGAAAATTTATTCTTGGTGG |
|  | pm4-R: **TTGCGTCGACTTAGAAACG**ATACAGACTAAATGGTTTGGCATCTAA |
|  | Vector5-F: **CGTTTCTAAGTCGACGCAA**AGCTTCTCGAGATCCGGCTGCTAA |
|  | Vector5-R: **TTGCCATCATATGGCTCTG**AAAATACAGGTTTTCGGGCCCC |

*^a^* All recombinant plasmids used in this study were constructed by homologous recombination using the ClonExpress multis One Step Cloning Kit.

*^b^* The homologous sequences are in bold.


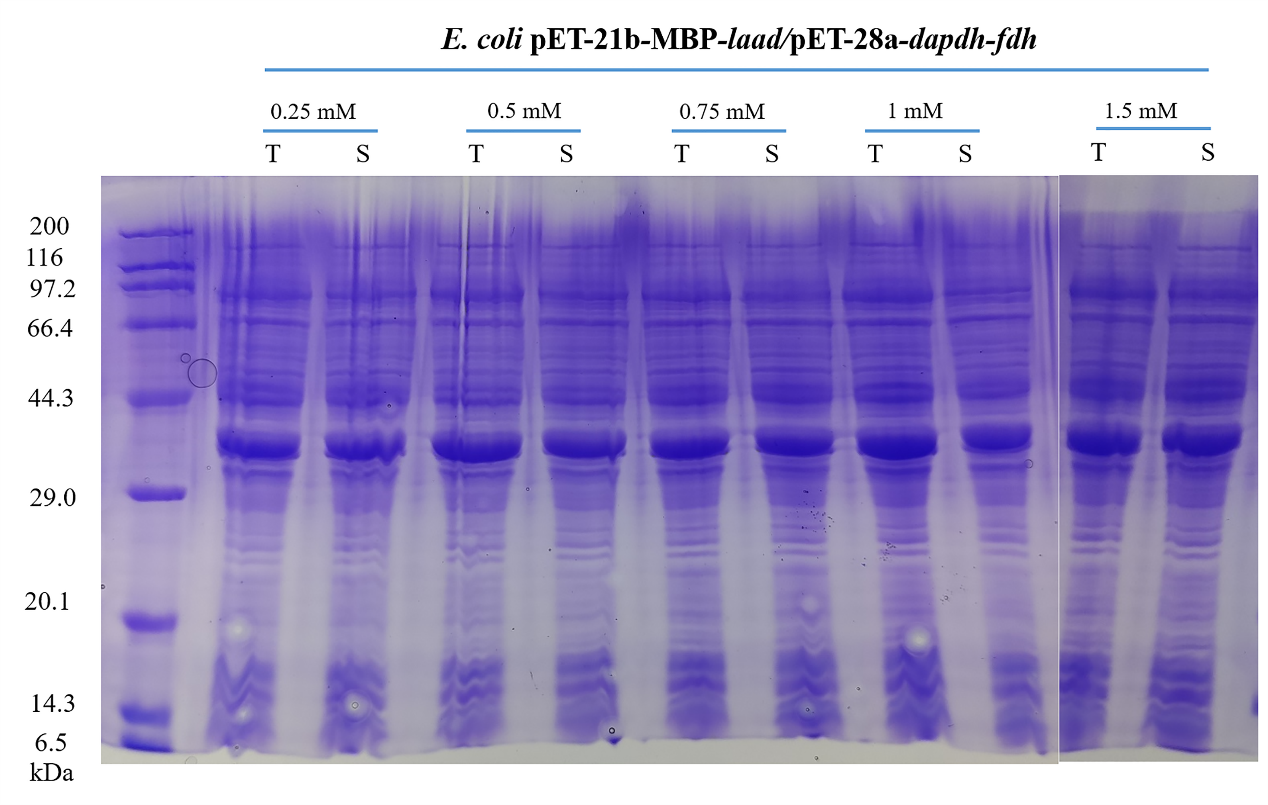


**Fig. S1** SDS-PAGE analysis of *E. coli* pET-21b*-*MBP*-laad/*pET-28a*-dapdh-fdh* induced by different concentrations of IPTG. T: total cell lysate; S: soluble fraction. The samples were generated from 50 mg/mL of *E. coli* pET-21b*-*MBP*-laad/*pET-28a*-dapdh-fdh* cells.


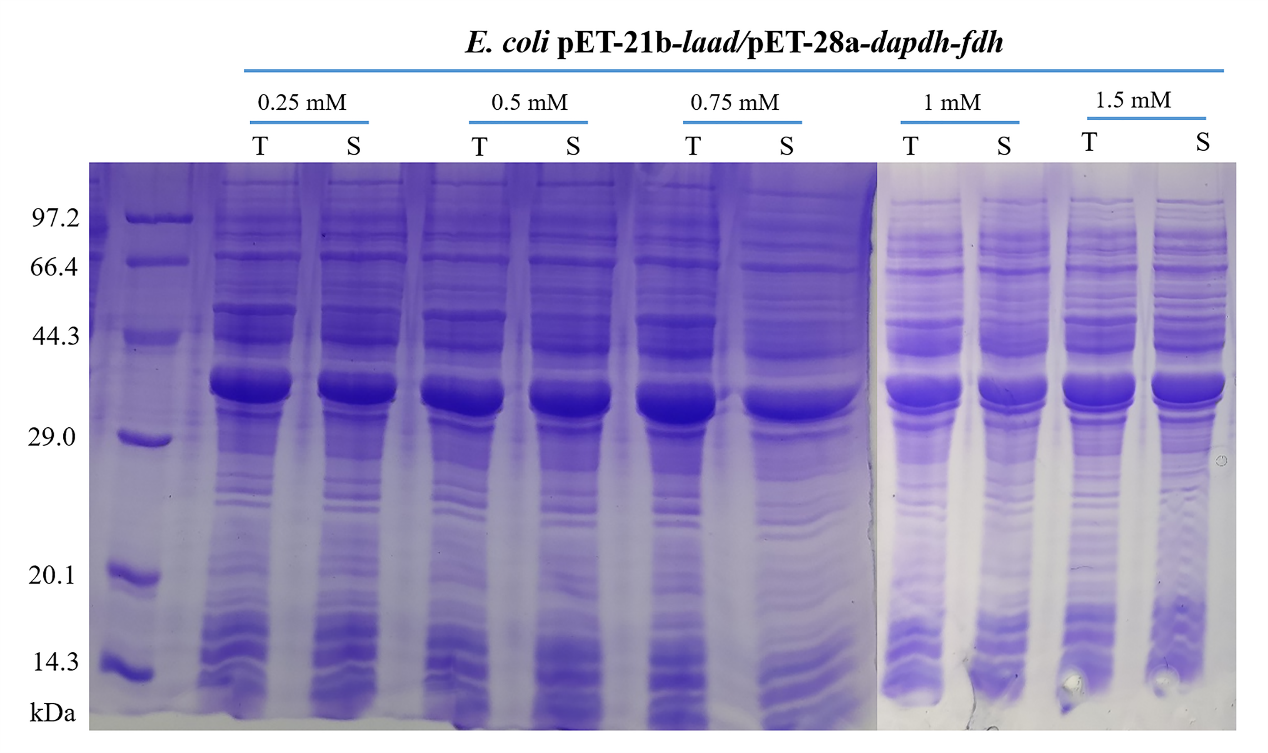


**Fig. S2** SDS-PAGE analysis of *E. coli* pET-21b*-laad/*pET-28a*-dapdh-fdh* induced by different concentrations of IPTG. T: total cell lysate; S: soluble fraction. The samples were generated from 50 mg/mL of *E. coli* pET-21b*-laad/*pET-28a*-dapdh-fdh* cells.


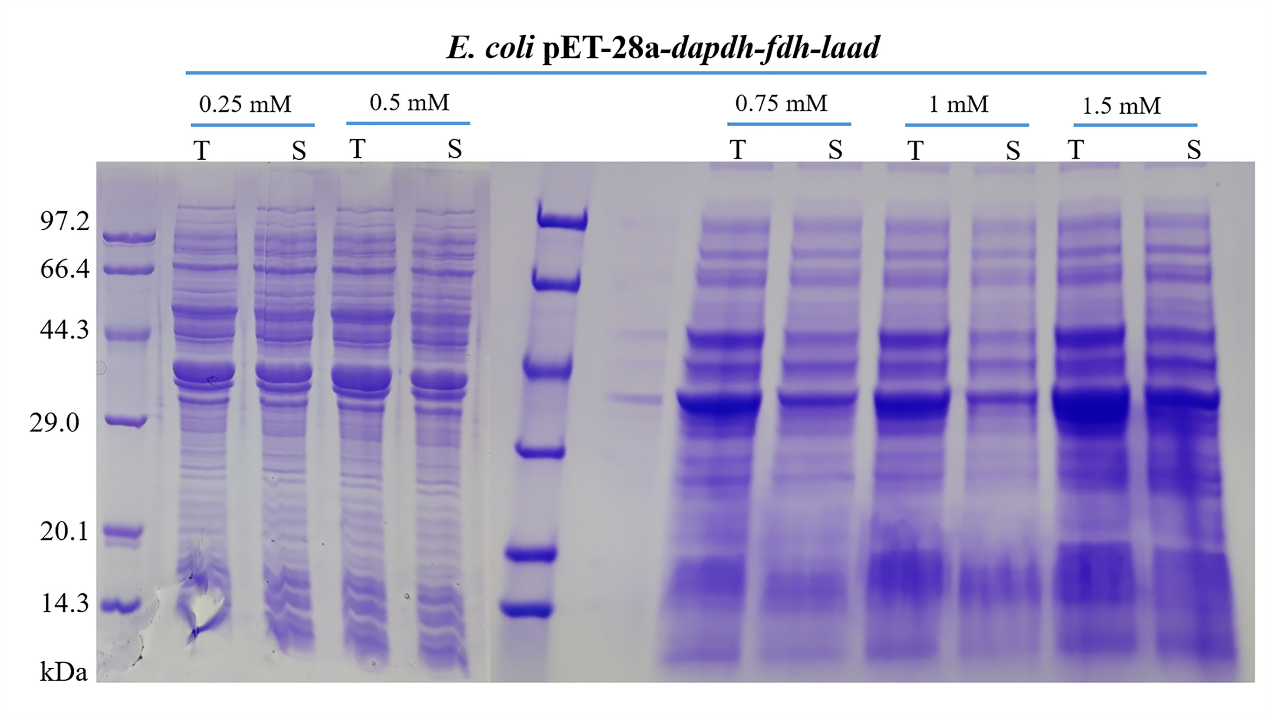


**Fig. S3** SDS-PAGE analysis of *E. coli* pET-28a*-dapdh-fdh-laad* induced by different concentrations of IPTG. T: total cell lysate; S: soluble fraction. The samples were generated from 50 mg/mL of *E. coli* pET-28a*-dapdh-fdh-laad* cells.


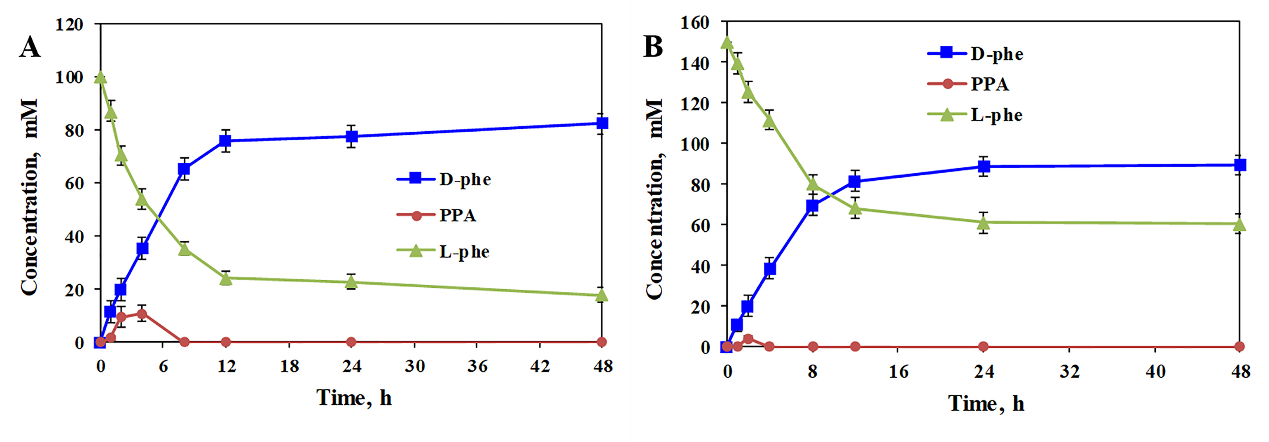


**Fig. S4** Time course of d-Phe production from diferent concentration of l-Phe. A: The concentration of l-Phe was 100 mM; B: The concentration of l-Phe was 150 mM. The concentration of *E. coli* pET-21b*-*MBP*-laad/*pET-28a*-dapdh-fdh* whole-cell biocatalyst was 75 mg/mL wet cell, the dosage of NADP^+^ was 0.1 time of substrate concentration, the dosage of NH_4_Cl was 4 times of the substrate concentration, and the dosage of sodium formate was 3 times of substrate concentration. All the reactions were carried out in Tris-HCl buffer (50 mM, pH 9.0) at 30 ℃ and 220 rpm.


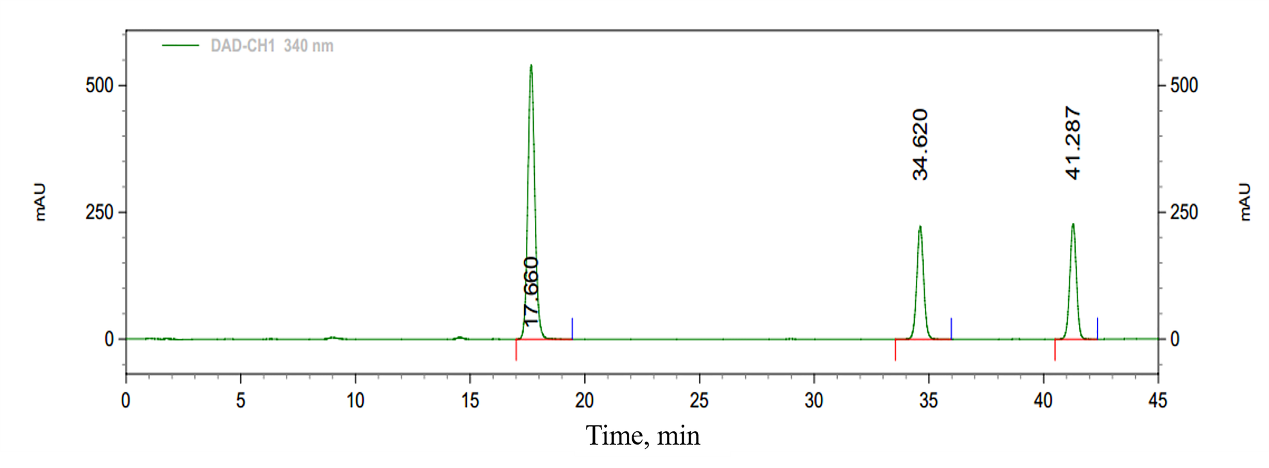


**Fig. S5** HPLC analysis of l-Phe and d-Phe. Retention time of l-Phe is 34.620 min; retention time of d-Phe is 41.287 min.


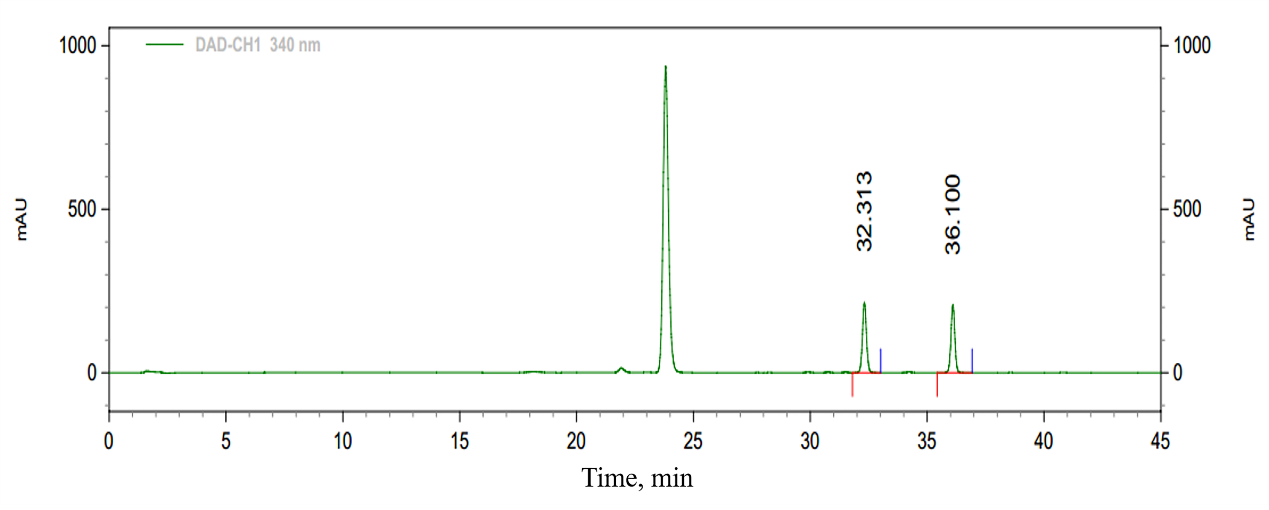


**Fig. S6** HPLC analysis of l-leucine and d-leucine. Retention time of l-leucine is 32.313 min; retention time of d-leucine is 36.100 min.


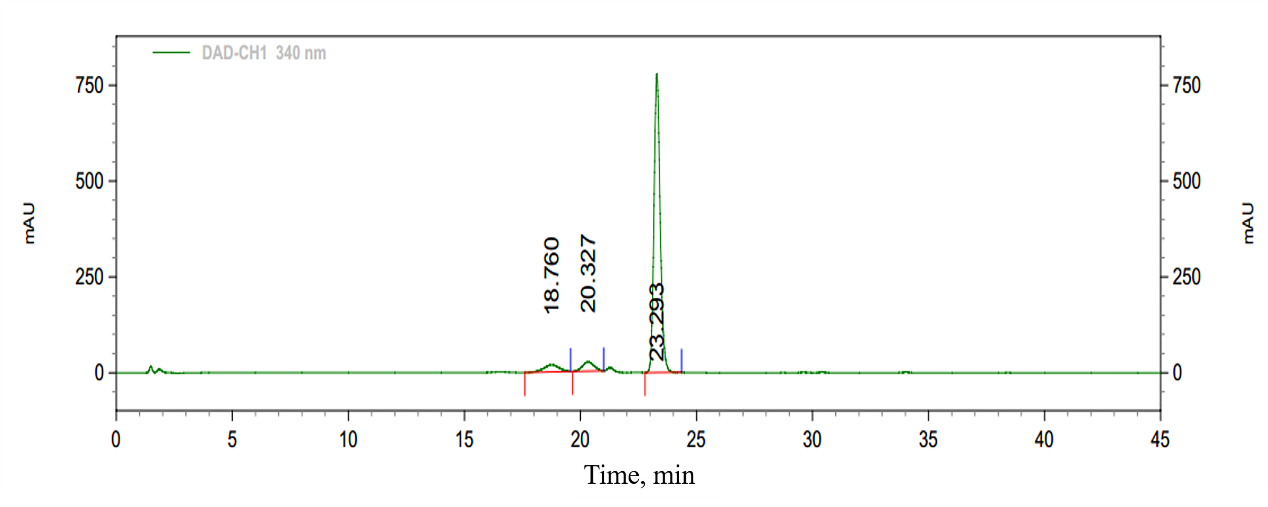


**Fig. S7** HPLC analysis of l-glutamic acid and d-glutamic acid. Retention time of l-glutamic acid is 18.760 min; retention time of d-glutamic acid is 20.327 min.


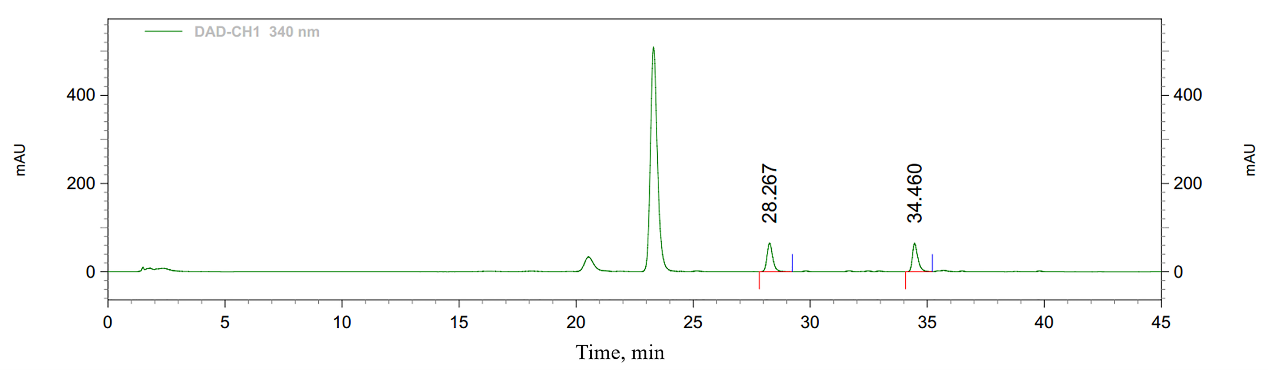


**Fig. S8** HPLC analysis of l-lysine and d-lysine. Retention time of l-lysine is 28.267 min; retention time of d-lysine is 34.46 min.


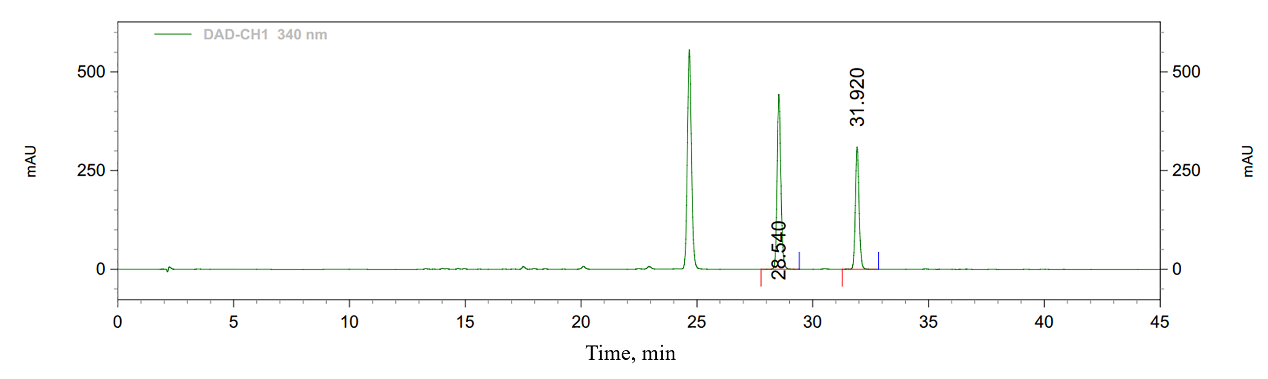


**Fig. S9** HPLC analysis of l-methionine and d-methionine. Retention time of l-methionine is 28.54 min; retention time of d-methionine is 31.92 min.


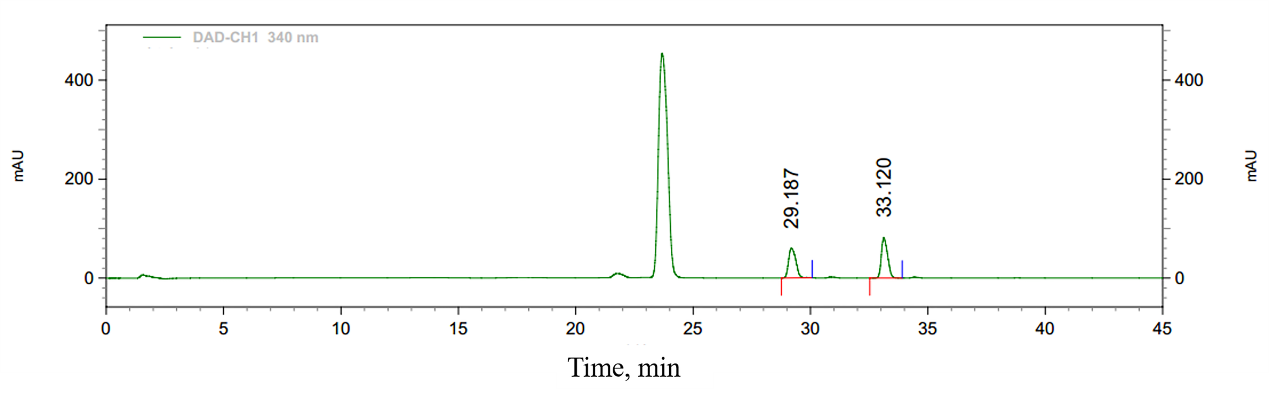


**Fig. S10** HPLC analysis of l-norvaline and d-norvaline. Retention time of l-norvaline is 29.153 min; retention time of d-norvaline is 33.120 min.


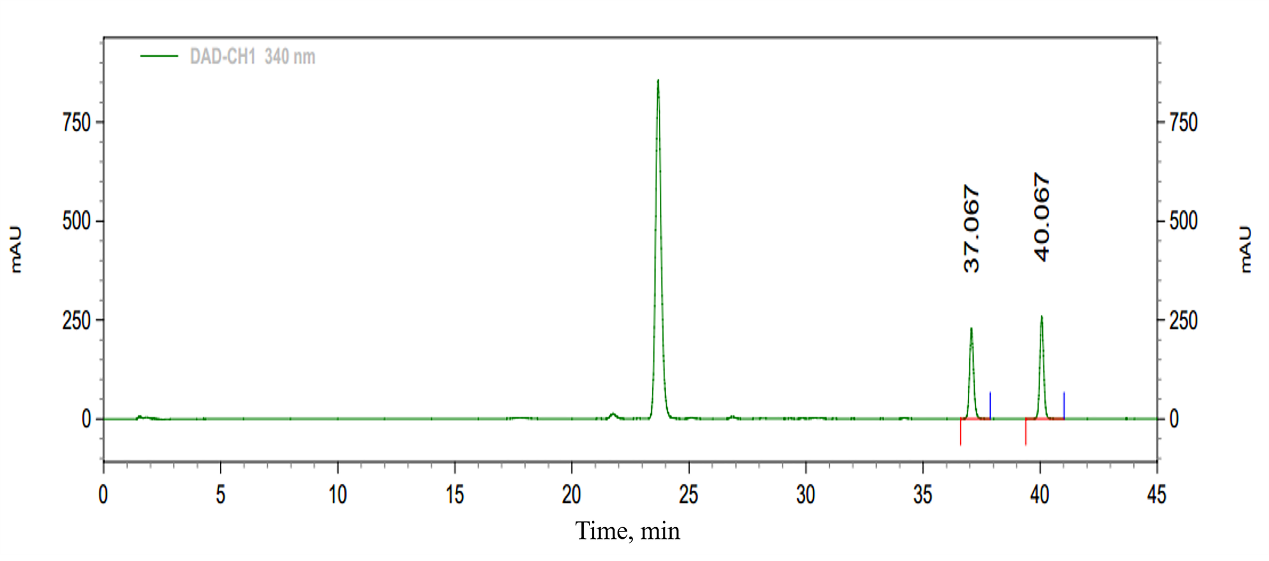


**Fig. S11** HPLC analysis of l-tyrosine and d-tyrosine. Retention time of l-tyrosine is 37.067 min; retention time of d-tyrosine is 40.067 min.


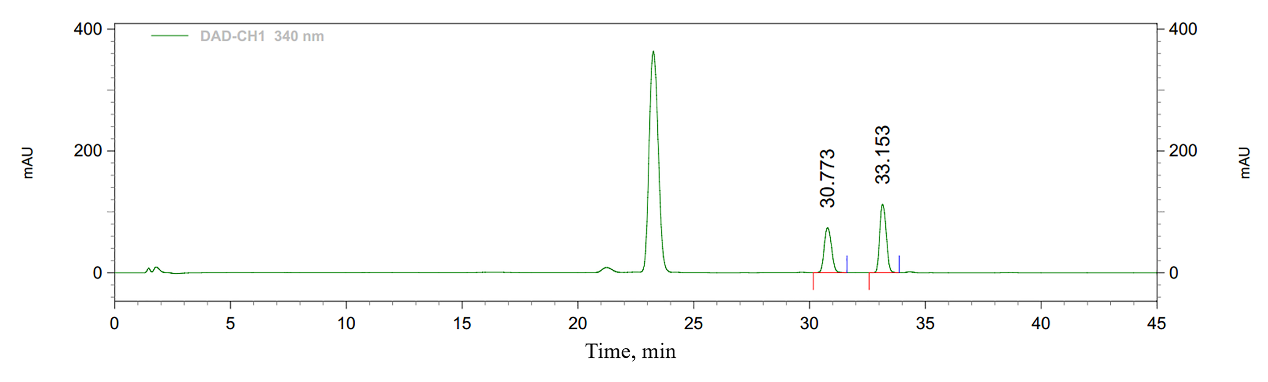


**Fig. S12** HPLC analysis of l-tryptophan and d-tryptophan. Retention time of l-tryptophan is 30.773 min; retention time of l-tryptophan is 33.153 min.


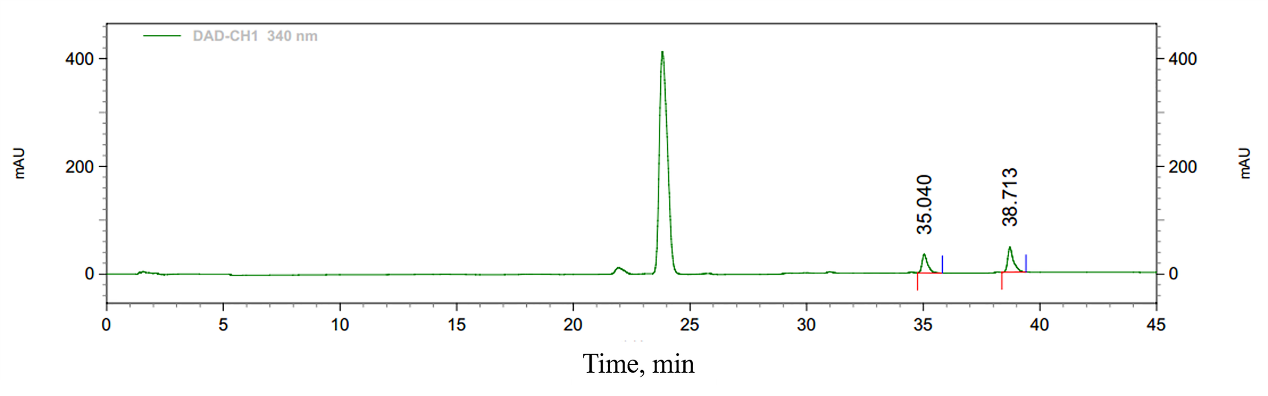


**Fig. S13** HPLC analysis of l-homophenyalanine and d-homophenyalanine. Retention time of l-homophenyalanine is 35.04 min; retention time of d-homophenyalanine is 38.713 min.


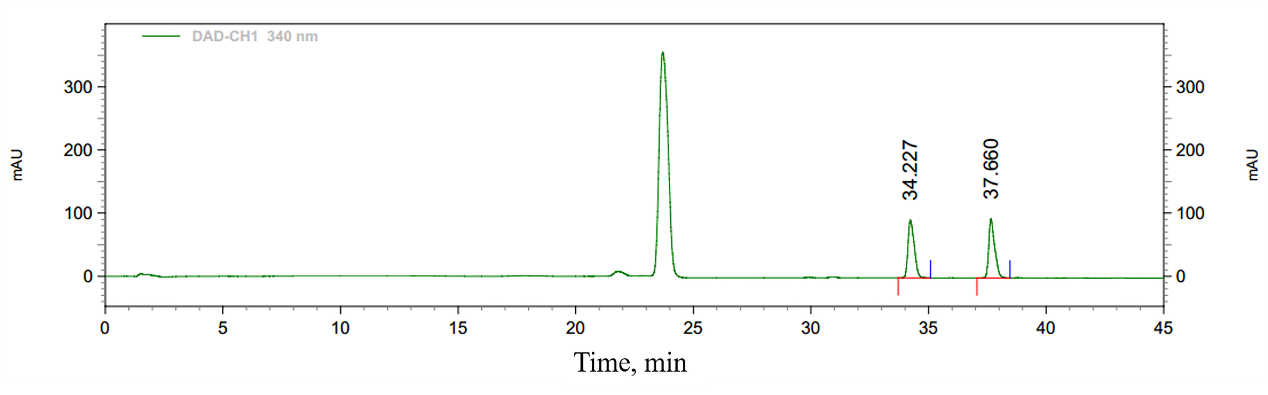


**Fig. S14** HPLC analysis of 2-chloro-l-phenylalanine and 2-chloro-d-phenylalanine. Retention time of 2-chloro-l-phenylalanine is 37.66 min; retention time of 2-chloro-d-phenylalanine is 34.227 min.


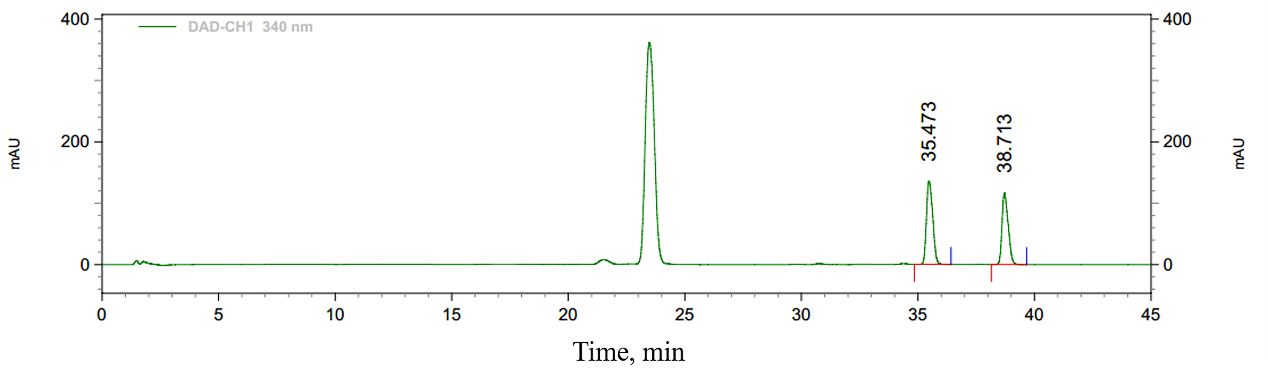


**Fig. S15** HPLC analysis of 3-chloro-l-phenylalanine and 3-chloro-d-phenylalanine. Retention time of 3-chloro-l-phenylalanine is 35.473 min; retention time of 3-chloro-d-phenylalanine is 38.713 min.


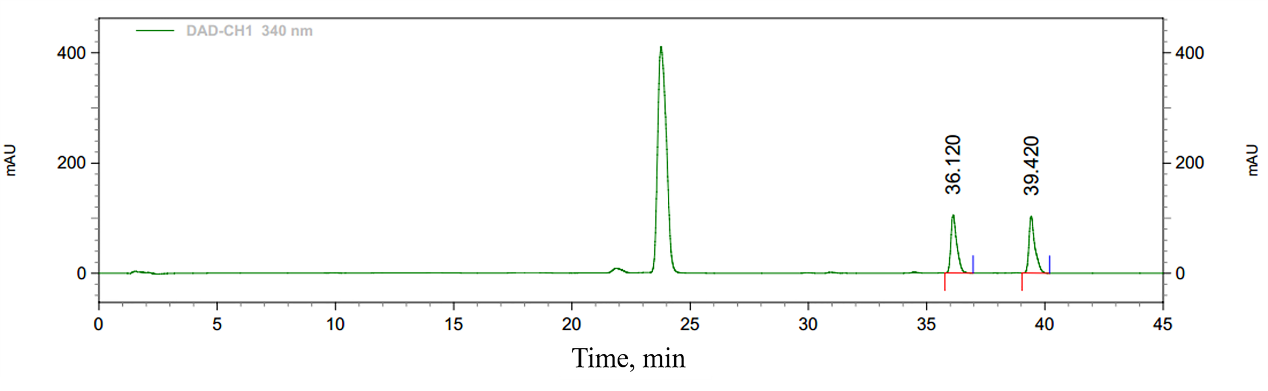


**Fig. S16** HPLC analysis of 4-chloro-l-phenylalanine and 4-chloro-d-phenylalanine. Retention time of 4-chloro-l-phenylalanine is 36.12 min; retention time of 4-chloro-d-phenylalanine is 39.42 min.


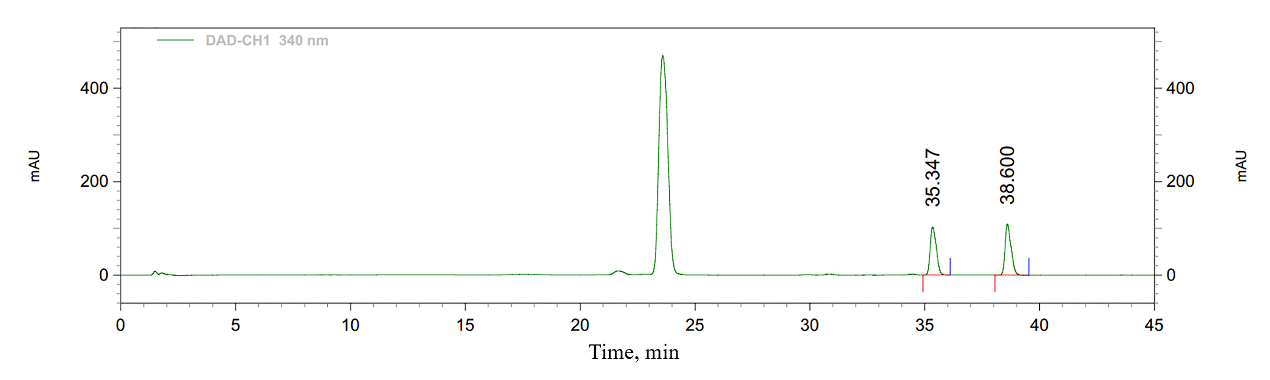


**Fig. S17** HPLC analysis of 4-methyl-l-phenylalanine and 4-methyl-d-phenylalanine. Retention time of 4-methyl-l-phenylalanine is 35.347 min; retention time of 4-methyl-d-phenylalanine is 38.600 min.
